# Supplementary material for: SpatialESD: Spatial Ensemble Domain Detection in Spatial Transcriptomics
Source: Adv Sci (Weinh). 2026 Feb 12;13(30):e20912. doi: 10.1002/advs.202520912 (PMC13248816; doi:10.1002/advs.202520912)
Supplement: Supplementary file 1 — Supporting File: advs74260‐sup‐0001‐SuppMat.docx. [file ADVS-13-e20912-s001.docx]

Supplementary Materials for

**SpatialESD: spatial ensemble domain detection in spatial transcriptomics**

Hongyan Cao*, Gaiqin Liu, Jingyi Xia, Runle Chen, Tong Wang, Xiaoling Yang, Ruiling Fang, Yanhong Luo, Ping Zeng, Hongmei Yu, Yanbo Zhang, Yuehua Cui*

*Corresponding author. Email: [caohy@sxmu.edu.cn](mailto:caohy@sxmu.edu.cn) and [cuiy@msu.edu](mailto:cuiy@msu.edu).

**This PDF file includes:**

Supplementary Text

Figs. S1 to S30

Tables S1 to S15

References (1 to 10)

**Supplementary Text**

**1.1 Supplementary Note 1: Application to the HER2 ST data**

We applied SpatialESD to an eight-slice HER2 dataset which was manually annotated by Andersson ^[1]^. The HER2 dataset was labeled into six categories: adipose tissue, cancer in situ, connective tissue, immune infiltrate, invasive cancer, and undetermined. In this analysis, we selected two base clustering methods, BASS and BayesSpace. The clustering performance of SpatialESD and the base methods was compared using the ARI, with detailed results presented in Table S4. The boxplot (Figure S11a) shows that SpatialESD achieved a slightly lower ARI value than BASS (by 0.016) but still outperformed other methods, exceeding them by 0.041 to 0.106. For spatial domain visualization analysis, we focused on slice C1, where the ARI value increased from the lowest base clustering result of 0.309 to 0.514 with SpatialESD, whereas the EnSDD ensemble approach resulted in a lower ARI of 0.297. Visualization results (Figure S11b-c) reveal that all base clustering methods performed poorly in identifying the invasive cancer region in the slice, particularly SpaGCN, which exhibited chaotic detection patterns. In contrast, SpatialESD consistently delivered superior visualization results compared to individual spatial domain detection methods as well as EnSDD.

Based on the identified spatial domains by SpatialESD, the enrichment results (Figure S11d) revealed significant enrichment of spatially differentially expressed genes in multiple cellular components and molecular functions. The enriched cellular components include the nucleus, cytosol, mitochondrion, mitochondrial matrix, cytoplasm, membrane, and nucleoplasm. The molecular functions primarily include protein binding, metal ion binding, and identical protein binding. KEGG pathway enrichment analysis showed that the spatially differentially expressed genes were significantly enriched in several key pathways, including metabolic pathways, cell cycle, human papillomavirus infection, RNA transport, cancer-related pathways, cell adhesion molecules (CAMs), p53 signaling pathway, human T-cell leukemia virus 1 infection, herpes simplex virus 1 infection, and proteoglycans in cancer. The visual domain results for the eight tissue sections are shown in Supplementary Figure S12.

**1.2 Supplementary Note 2: Application to the Invasive Ductal Carcinoma ST data**

We also performed corresponding trajectory analysis, where Figure S13a reveals the developmental direction from healthy tissue to tumor tissue, showing the developmental process of tumor cells and their interactions with immune cells in the tumor microenvironment.

Additionally, spatial cell-cell communication analysis was conducted (Figures S13b-e), where we integrated the ST data with the single-cell reference dataset HumanPrimaryCellAtlasData to explore the interactions between different cell types within the tissue (Figures S13c). The annotation results included seven cell types: B cells, endothelial cells, epithelial cells, macrophages, monocytes, T cells, and tissue stem cells. The analysis revealed significant spatial communication between tumor cells and immune cells, particularly in the tumor microenvironment, as well as interactions between tumor cells and stromal cells. Notably, we observed that Macrophage showed strong signaling interactions with B-cell (Figure S13b), which is consistent with previous research findings ^[2,3]^. Figure S13d shows that there is a strong signaling connection between B cells, endothelial cells, and epithelial cells, reflected by the thicker connection lines between these cell types, indicating a strong interaction in their signaling pathways. In contrast, T cells, macrophages, and tissue stem cells show weaker connections with the above cell types, with thinner connection lines, suggesting less or weaker signaling interaction between these cells. Notably, there is no significant interaction between monocytes and other cell types, indicating that B cells, endothelial cells, and epithelial cells may play a more critical role in immune responses, cell migration, and other biological processes within the tissue, while their interactions with other cell types are relatively limited. Overall, this result suggests that B cells, endothelial cells, and epithelial cells may closely cooperate through a cellular communication network under specific physiological or pathological conditions, potentially playing an important role in immune responses and the tumor microenvironment. Figure S13d shows that the dark color of epithelial cells in all roles indicates their central role in the cell communication network, suggesting that they play an important role in signaling transmission, reception, mediation, and influence, particularly in processes such as immune response, cell migration, and tumor progression ^[4,5]^. The first two plots in Figure S13e show the spatial distribution of TGFB1 (ligand) and TGFBR1 (receptor) within the tissue, with a red gradient indicating the expression intensity of these molecules in different regions. The deeper the red, the stronger the expression. This result suggests the activity and spatial localization of TGFB1 and TGFBR1 within the tissue. The third plot further illustrates the expression of the TGFB1-TGFBR1-TGFBR2 ligand-receptor pair, specifically showing the signaling interaction between TGFB1 (ligand) and TGFBR1 and TGFBR2 (receptors). This plot allows us to observe the expression intensity and spatial distribution of these ligand-receptor pairs in the tissue, with deeper colors indicating stronger expression. It helps reveal the critical role of these molecules in cell-cell communication, especially their potential involvement in processes like immune response and tumor progression. This highlights the dynamic cell-cell interactions that shape the spatial architecture of the tumor and its surrounding microenvironment, providing valuable insights into the mechanisms of tumor progression and immune evasion. The complete results of the downstream analysis are provided in Supplementary Figures S14-S17.

**1.3 Supplementary Note 3: Application to the Ovarian Cancer ST data**

Trajectory analysis (Figure S19a) indicated migration or infiltration of CD45-labeled immune cells into other regions, suggesting their dynamic role in shaping tumor progression and immune responses within the TME.

Additionally, we performed spatial cell-cell communication analysis (Figures S20 and S21), integrating spatial transcriptomic data with a single-cell reference dataset (Figure S19b) to explore interactions among distinct cell types within the tissue. The annotated cell types included seven categories: erythroblasts, endothelial cells, epithelial cells, macrophages, monocytes, neurons, and tissue stem cells. The analysis of communication quantity (Figure S20, upper) and interaction strength (Figure S20, lower) revealed that erythroblasts exhibited the highest number of interactions, suggesting their frequent cell-cell communication within the local microenvironment, while macrophages displayed the strongest interaction strength, implying stable or intense signal exchange potentially related to inflammatory signaling, phagocytic regulation, or immune microenvironment shaping. Erythroblasts exhibited the highest number of cell-cell interactions, highlighting their active communication within the TME. Macrophages displayed robust signal exchange, underscoring their pivotal role in tumor immunity, particularly tumor-associated macrophages (TAMs) in driving tumor progression and immune evasion ^[6]^.

For outgoing communication patterns (Figure S21a), both the Cophenetic and Silhouette values showed abrupt declines when the number of output patterns reached 6. Cell pattern analysis (Figure S21b, left) demonstrated high similarity in communication patterns across different cell types, with closely branched structures, indicating shared signaling characteristics. Contribution analysis (Figure S21b, right) revealed distinct cell-type dominance in specific patterns: endothelial cells were most active in Pattern 6, epithelial cells enriched in Pattern 4, macrophages concentrated in Pattern 2, and erythroblasts dominated Pattern 1. Communication pattern analysis highlighted pathway-specific contributions: the CALCR pathway dominated Pattern 4 (darkest color), ANGPTL, ILT, and SLURP pathways contributed most to Pattern 2, while PTN, PARs, and VEG pathways played central roles in Pattern 1. Cellular pattern analysis revealed shared signaling features among distinct cell types, with endothelial cells predominantly contributing to Pattern 6, epithelial cells to Pattern 4, macrophages to Pattern 2, and erythroblasts to Pattern 1. Pathway-specific dominance was observed across patterns: CALCR signaling dominated Pattern 4 (epithelial cells), ANGPTL, ILT, and SLURP pathways in Pattern 2 (macrophages), and PTN, PARs, and VEG pathways in Pattern 1 (erythroblasts), implicating these pathways in angiogenesis and immune regulation. These findings provide novel insights into the cellular communication mechanisms within the tumor immune microenvironment, offering a theoretical foundation for future immunotherapeutic strategies. A River plot (Figure S21b) visually summarized the distribution of cell types across communication patterns and the pathway contributions within each pattern. The complete results of the downstream analysis are provided in Supplementary Figures S22-S26.

**1.4 Supplementary Note 4: Application to the Stereo-seq data**

We applied SpatialESD to the single-cell Stereo-seq dataset of the axolotl telencephalon from Wei et al. ^[7]^. This dataset provides transcriptomic information at subcellular resolution, which was aggregated to single-cell precision, comprising 4,410 cells across 17 annotated cell types. We benchmarked SpatialESD against five spatial clustering methods using default parameters, and assessed clustering performance using the ground-truth cell type annotations with the ARI. As shown in Table S13, SpatialESD achieved the highest ARI of 0.678, outperforming all baseline methods, as well as the ensemble method EnSDD (0.665) constructed from the same base clustering.

Qualitative visualization (see Figure S28) further exhibits the superior performance of SpatialESD in distinguishing rare and spatially restricted cell populations. For example, developing Ependymoglial Cells (dEGCs) were resolved as a distinct, continuous cluster that aligns closely with the original biological annotations (see Figure S28). In contrast, most baseline methods produced fragmented or noisy clusters that failed to accurately capture the spatial niche of these progenitor cells.

**1.5 Supplementary Note 5: Application to the Slide-seqV2 data**

We applied SpatialESD to a high-resolution Slide-seqV2 mouse embryo dataset ^[8]^, which contains 8,425 beads across 16 annotated cell types, with a spatial resolution of approximately 10 µm. Following common practice in previous studies ^[9,10]^, we used the number of annotated cell types as a reference clustering number to guide spatial domain identification. We applied SpatialESD and five base methods to infer spatial domains and evaluated the results both quantitatively and qualitatively. Quantitative assessment (see Table S13) was performed by comparing inferred domains with the known cell-type annotations using the ARI. Although BASS achieved a slightly higher ARI in this dataset, SpatialESD maintained highly competitive performance while providing superior spatial continuity.

Qualitative visualization (see Figure S29) further highlights the advantages of SpatialESD. While individual methods such as BayesSpace and STAGATE show significant “salt-and-pepper” noise due to high data sparsity, SpatialESD effectively filters out local artifacts through ensemble learning, producing smoother and more anatomically plausible spatial domains. In contrast, methods like SpatialPCA tend to over-smooth the data, which can obscure fine-grained local features. By leveraging a consensus mechanism across multiple base algorithms, SpatialESD achieves a balance between preserving biological detail and ensuring spatial continuity, avoiding biases or failure modes associated with any single method.

**1.6 Supplementary Note 6: Sensitivity analysis of the random walk length** $\boldsymbol{r}$

In SpatialESD, the random walk length $r$ controls the scale of structural information captured from the cluster-wise similarity graph. Specifically, different step lengths of the random walk encode graph structure at different resolutions, where shorter walks emphasize local relationships and longer walks progressively incorporate more global structural information. By aggregating random walk transition probabilities from step 1 to $r$, SpatialESD integrates multi-scale structural patterns to refine the cluster-wise similarity matrix.

To evaluate the sensitivity of SpatialESD to the choice of the random walk length, we conducted a systematic sensitivity analysis by varying $r$ over a broad range of values $r=5,10,15,20,25,30,35,40$. The analysis was performed on two representative datasets with distinct structural characteristics: the DLPFC brain dataset, which exhibits hierarchical tissue organization, and a breast cancer dataset, which represents tumor tissue with more heterogeneous spatial patterns.

The results are shown in Figure S30. SpatialESD exhibits stable clustering performance across a wide range of random walk lengths $r$ on the DLPFC and breast cancer datasets. In particular, using a moderately larger value of $r$ (e.g., $r>10$) leads to improved clustering performance compared with very small values. Moreover, intermediate values of $r$ (approximately between 15 and 20) consistently yield stable and relatively high ARI scores across different tissue sections. Among the tested values, $r=20$ achieves the highest median ARI and is therefore used as the default setting in our experiments.

**Reference**

1. Andersson A, Larsson L, Stenbeck L, et al. Spatial deconvolution of HER2-positive breast cancer delineates tumor-associated cell type interactions. *Nature Communications*, 2021, 12(1): 6012.

2. Iliopoulou M, Bajur A T, Mcarthur H C, et al. Extracellular matrix rigidity modulates physical properties of subcapsular sinus macrophage-B cell immune synapses. *Biophysical Journal*, 2024, 123(15): 2282-2300.

3. Nyström H, Jönsson M, Nilbert M, et al. Immune-cell infiltration in high-grade soft tissue sarcomas; prognostic implications of tumor-associated macrophages and B-cells. *Acta Oncologica*, 2023, 62(1): 33-39.

4. Manfioletti G, Fedele M. Epithelial–Mesenchymal Transition (EMT). *International Journal of Molecular Sciences*, 2023: 11386.

5. Liang L, Kaufmann A M. The significance of cancer stem cells and epithelial–mesenchymal transition in metastasis and anti-cancer therapy. *International Journal of Molecular Sciences*, 2023, 24(3): 2555.

6. Noy R, Pollard J W. Tumor-associated macrophages: from mechanisms to therapy. *Immunity*, 2014, 41(1): 49-61.

7. Wei X, Fu S, Li H, et al. Single-cell Stereo-seq reveals induced progenitor cells involved in axolotl brain regeneration. *Science*, 2022, 377(6610): eabp9444.

8. Sampath Kumar A, Tian L, Bolondi A, et al. Spatiotemporal transcriptomic maps of whole mouse embryos at the onset of organogenesis. *Nature Genetics*. 2023;55(7):1176-1185.

9. Xu C, Jin X, Wei S, et al. DeepST: identifying spatial domains in spatial transcriptomics by deep learning. *Nucleic Acids Research*, 2022, 50(22): e131-e131.

10. Kang L, Zhang Q, Qian F, et al. Benchmarking computational methods for detecting spatial domains and domain-specific spatially variable genes from spatial transcriptomics data. *Nucleic Acids Research*, 2025, 53(7): gkaf303.

**Supplementary Figures:**


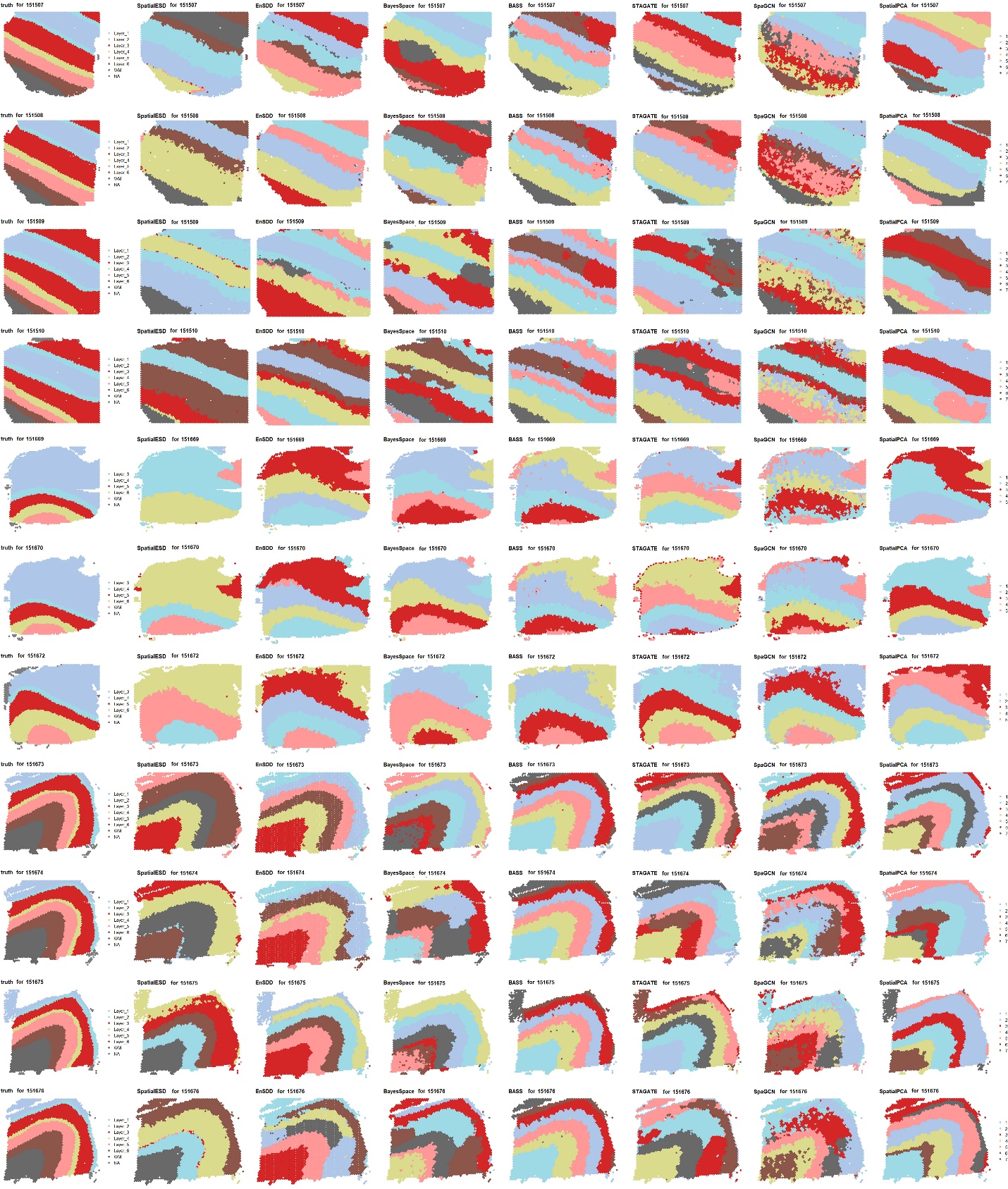


**Figure S1.** Spatial domain visualization results for different methods on 12 slices of the Human Dorsolateral Prefrontal Cortex Dataset.


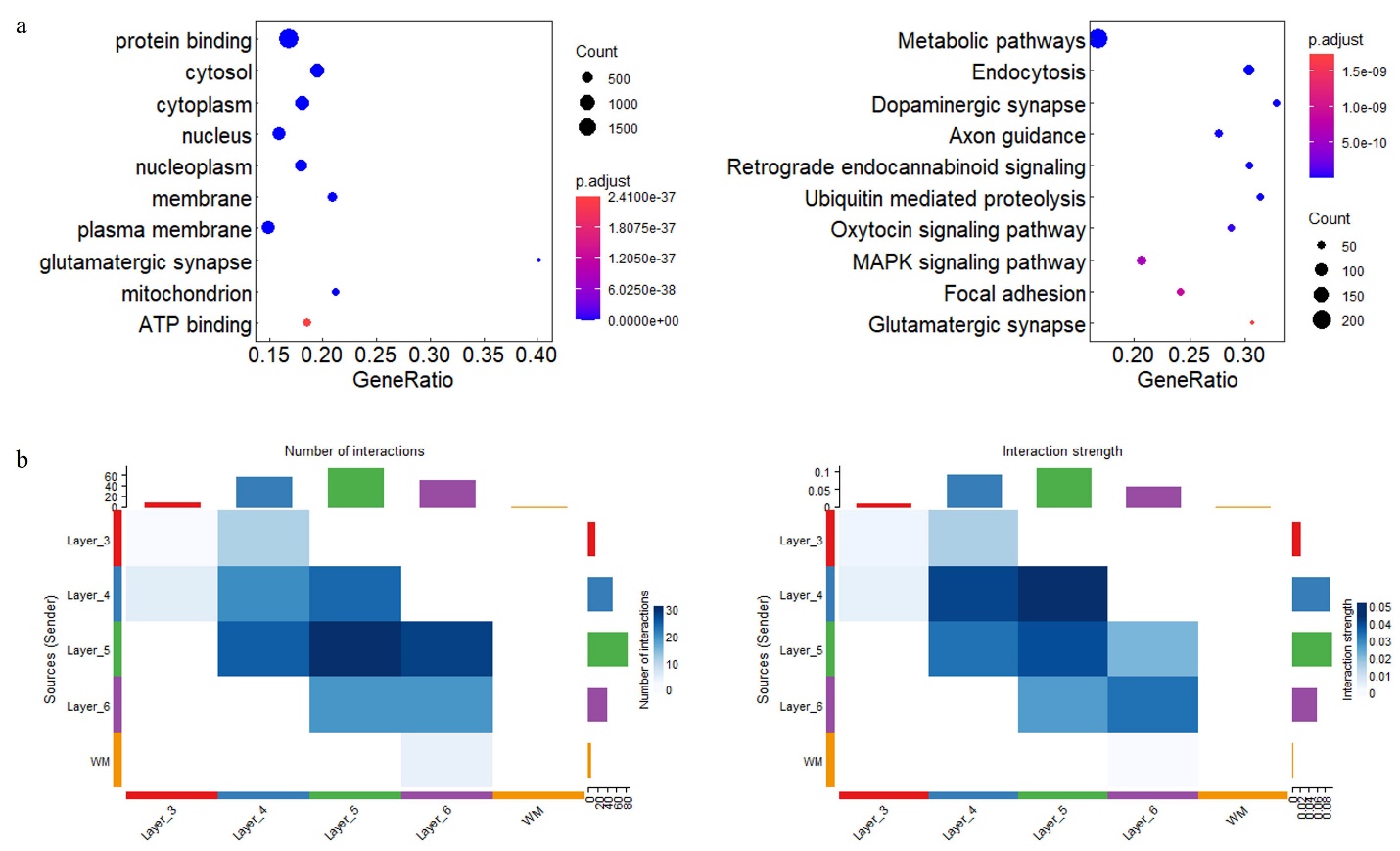


**Figure S2.** (a) Enrichment Analysis of Slice 151671 in DLPFC: Left: GO Analysis, Right: KEGG Analysis. (b) Number of Interactions and Interaction Strength.


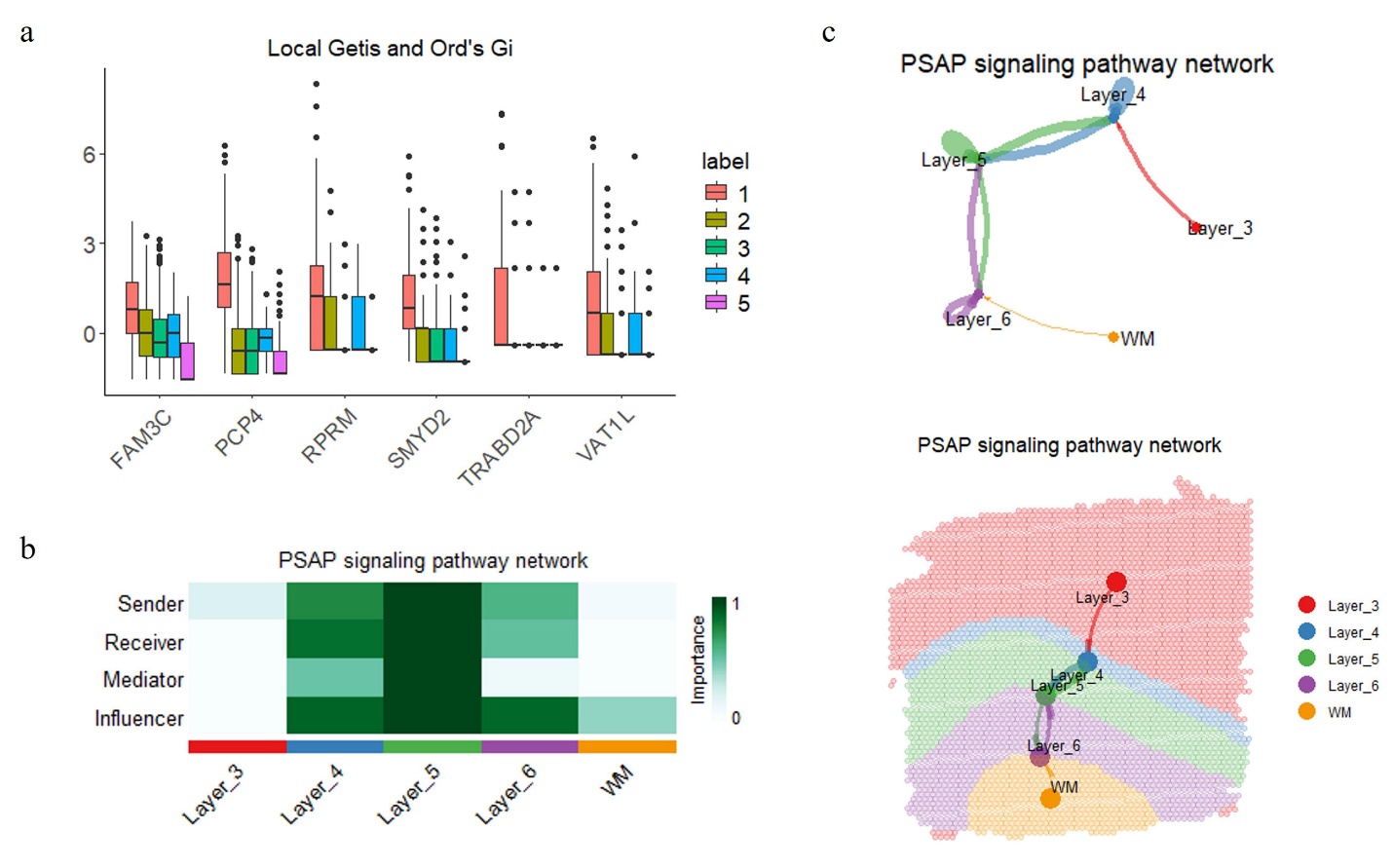


**Figure S3.** (a) Expression levels of differentially expressed genes in different spatial domains based on spatial autocorrelation analysis for 151671. (b) Heatmap of Centrality Scores in PSAP Signaling Pathway Network for 151671. (c) Circle plot and spatial map with signaling overlay of the PSAP Signaling Pathway Network for 151671.


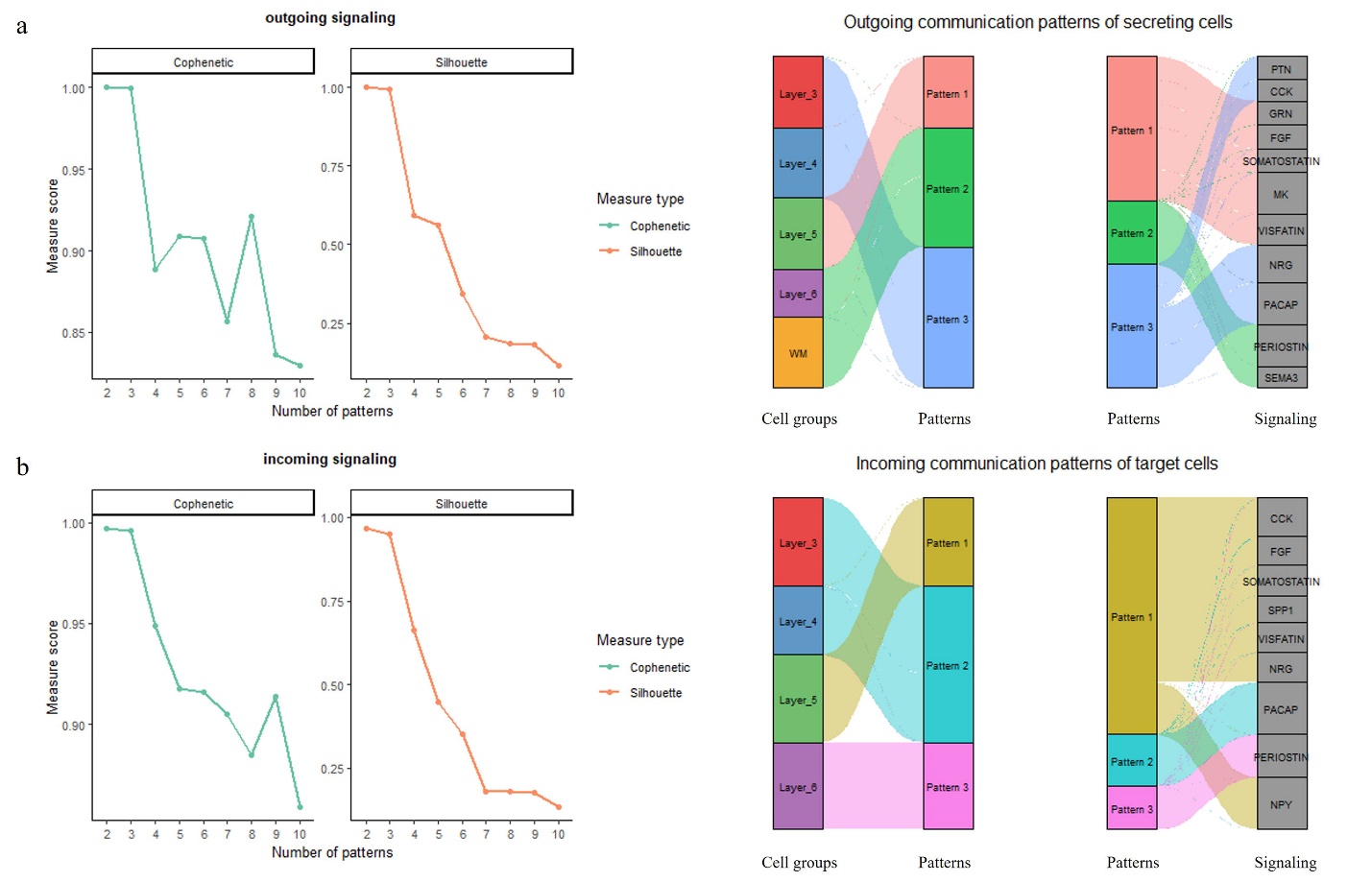


**Figure S4.** (a) Outgoing Communication Patterns in Slice 151671. Left: Determination of the number of inferred outgoing communication patterns; Right: River plot showing outgoing communication patterns of secreting cells. (b) Incoming Communication Patterns in Slice 151671. Left: Determination of the number of inferred incoming communication patterns; Right: River plot showing incoming communication patterns of secreting cells.


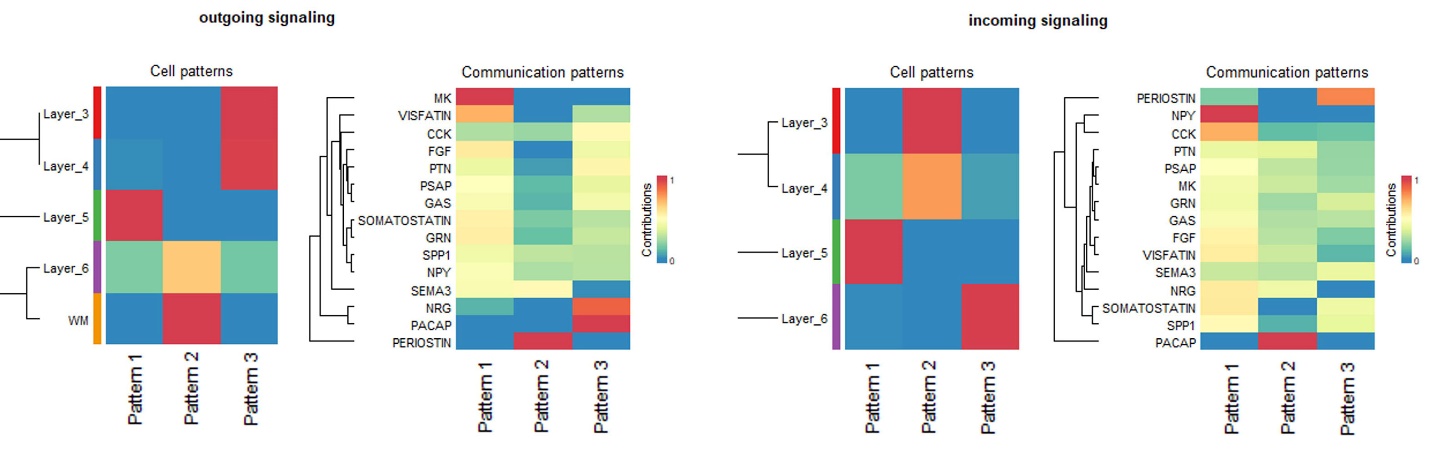


**Figure S5.** Cell and Communication Pattern Contributions in Outgoing and Incoming Signaling for 151671.


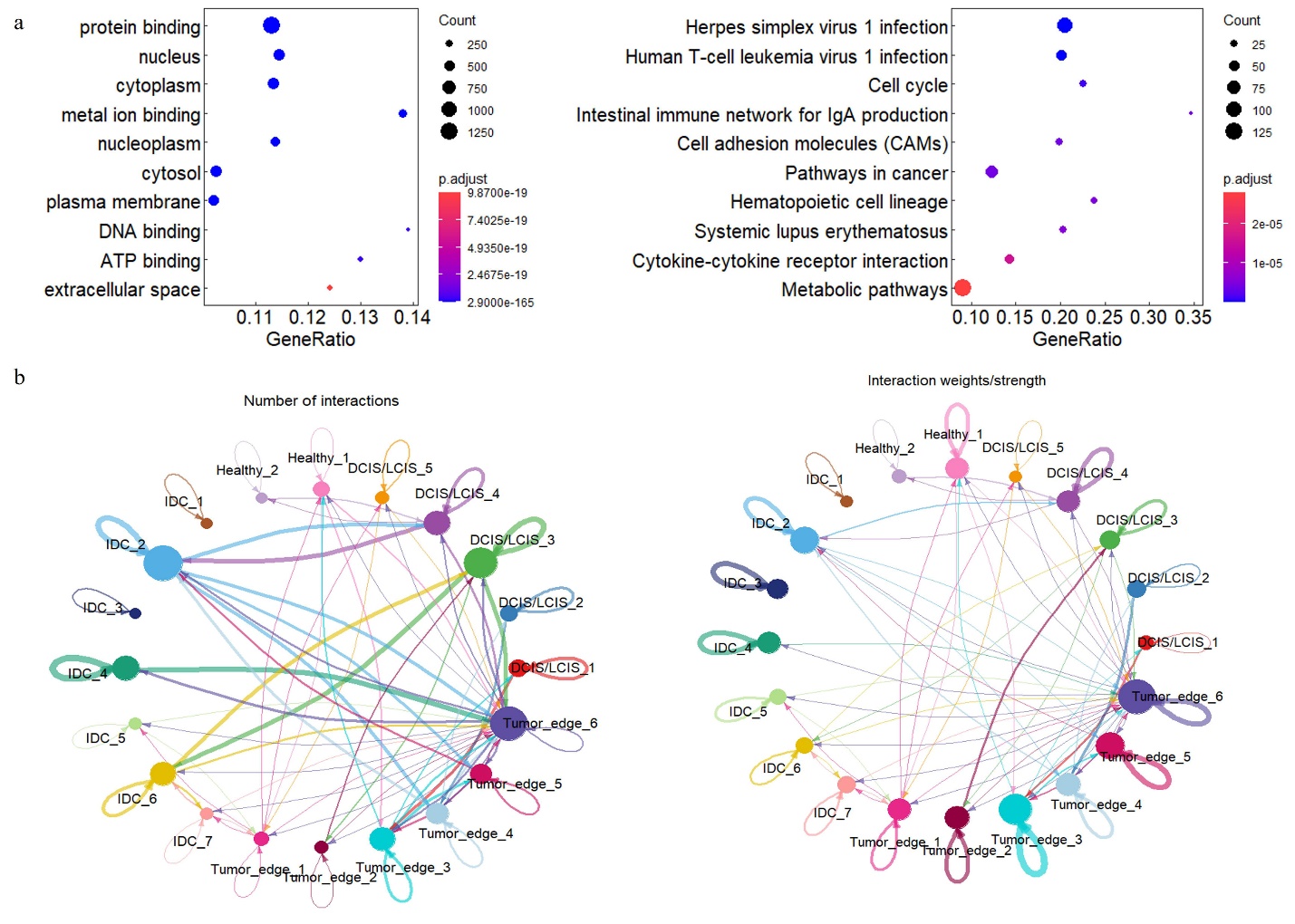


**Figure S6.** (a) Enrichment Analysis of breast cancer: Left: GO Analysis; Right: KEGG Analysis. (b) Number of Interactions and Interaction Strength of breast cancer.


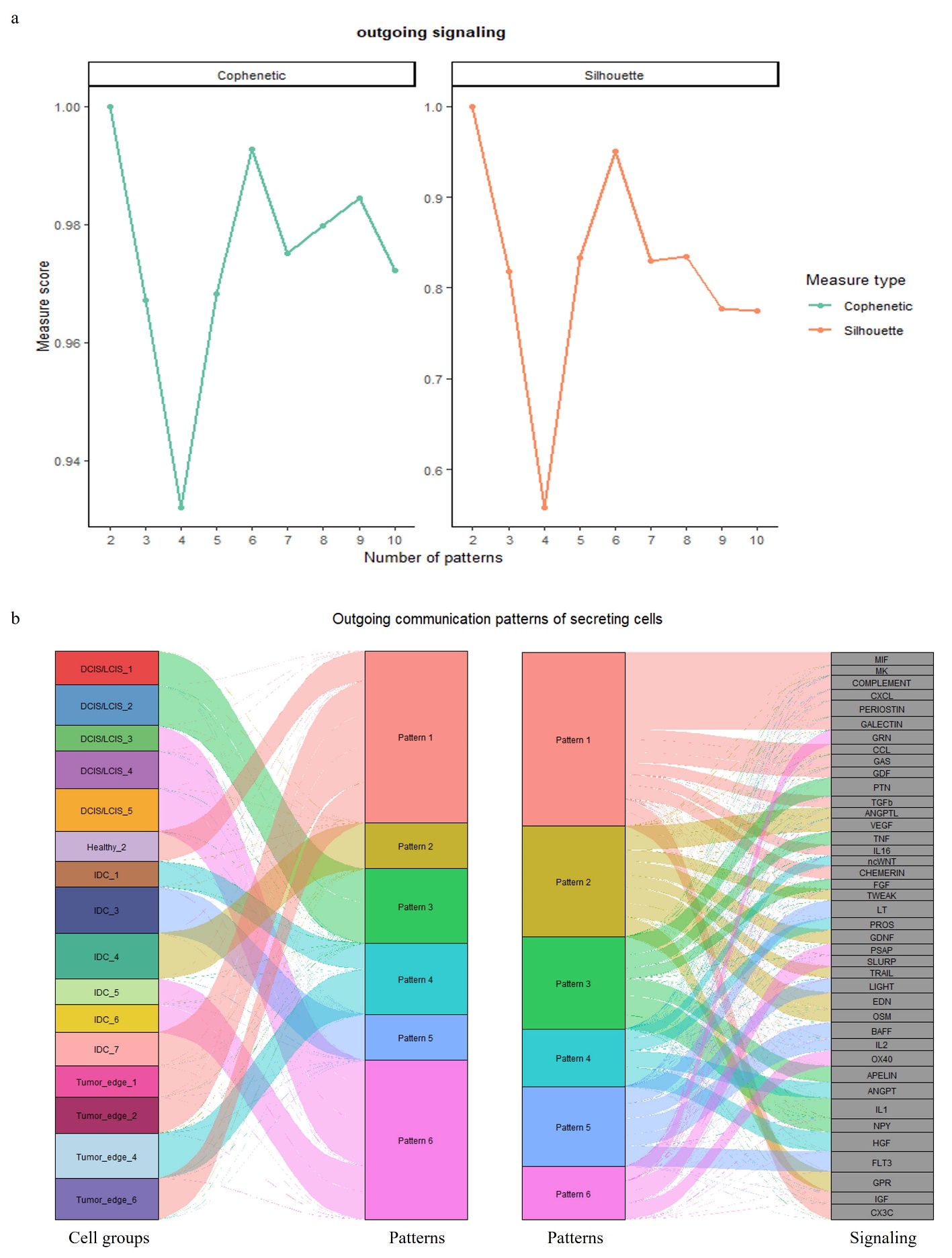


**Figure S7.** (a) Determination of the number of inferred outgoing communication patterns for breast cancer. (b) River plot showing outgoing communication patterns of secreting cells.


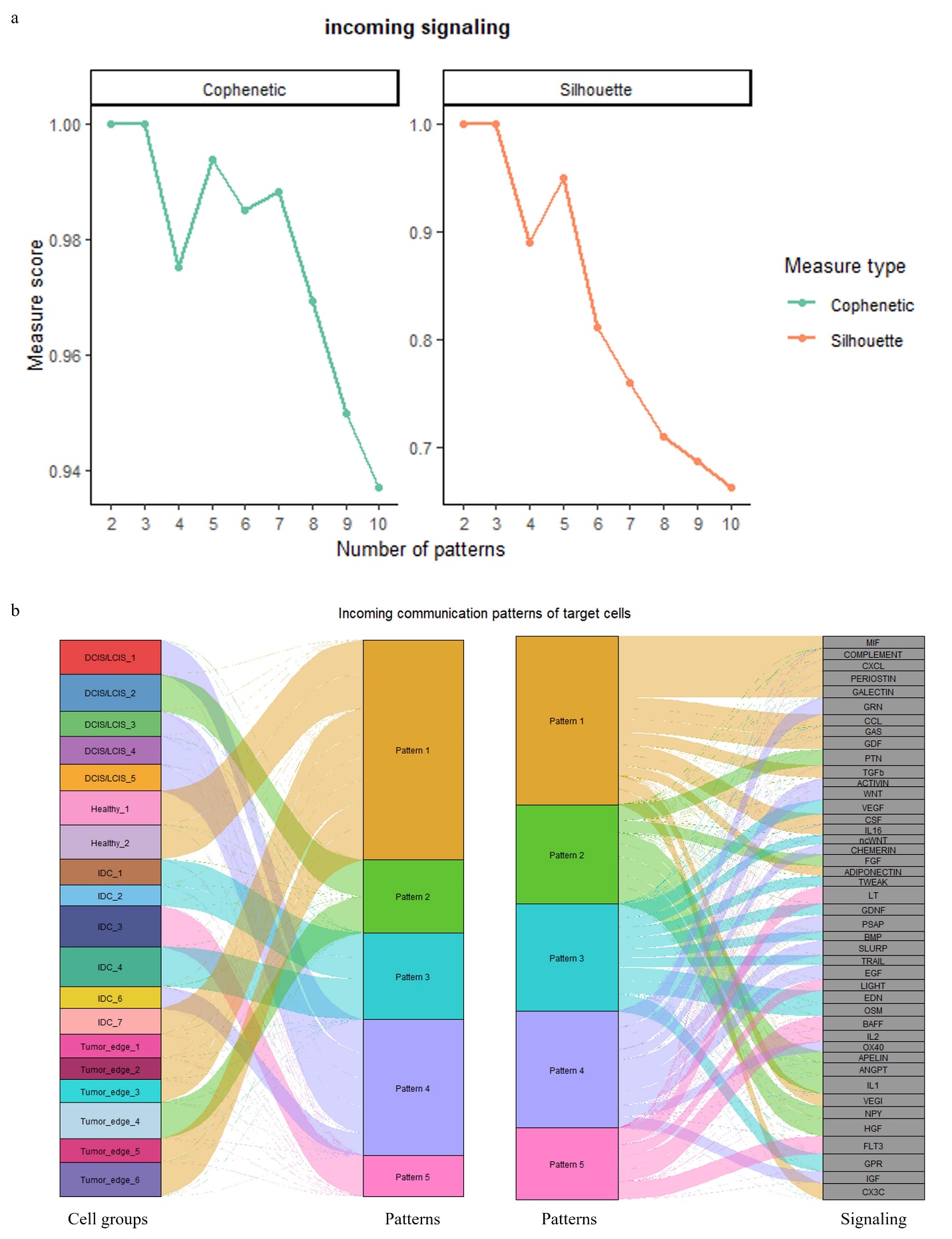


**Figure S8.** (a) Determination of the number of inferred incoming communication patterns for breast cancer. (b) River plot showing incoming communication patterns of secreting cells.


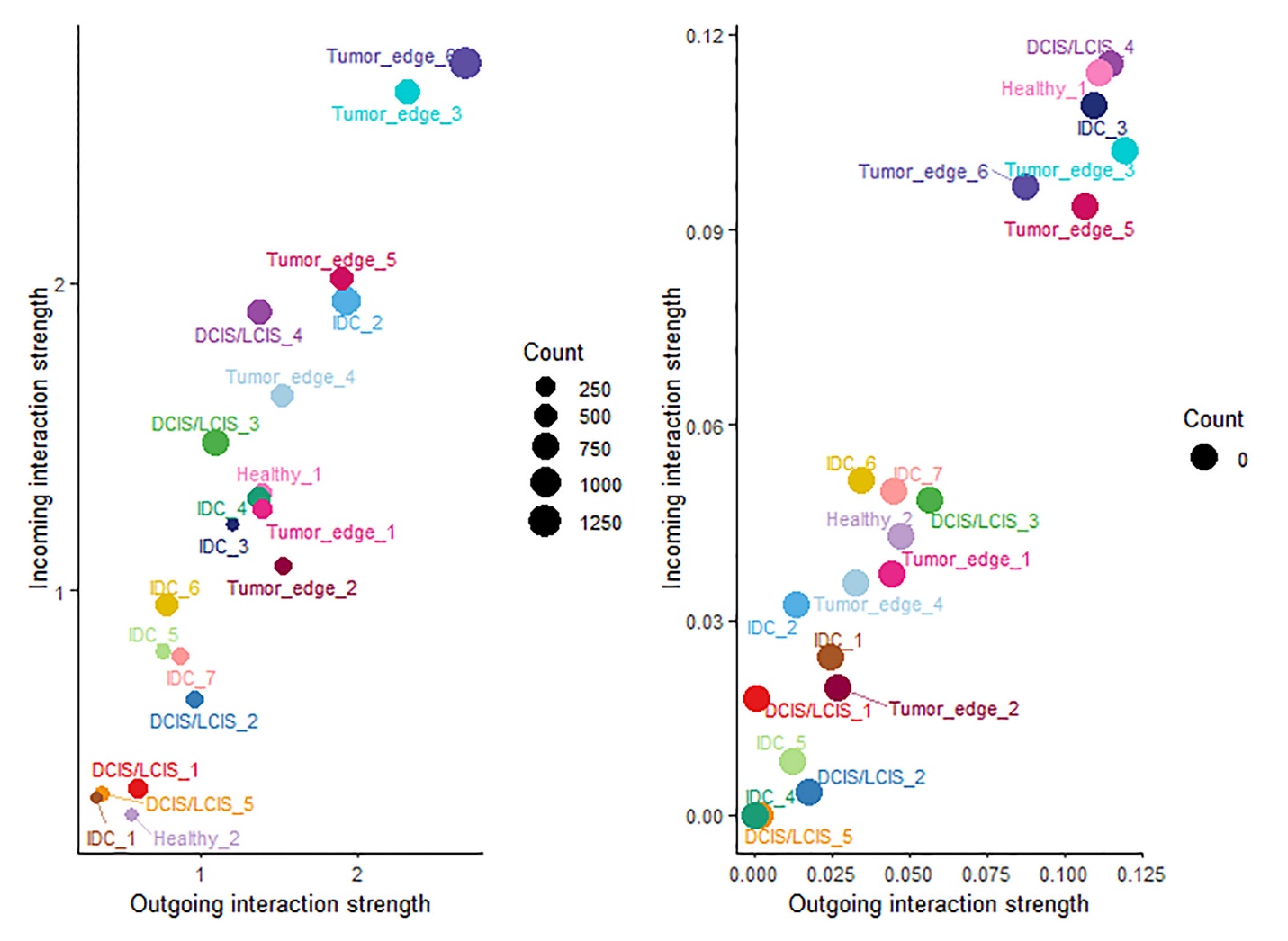


**Figure S9.** Global and CXCL Specific Signaling Role Analysis in Cell-Cell Communication for breast cancer.


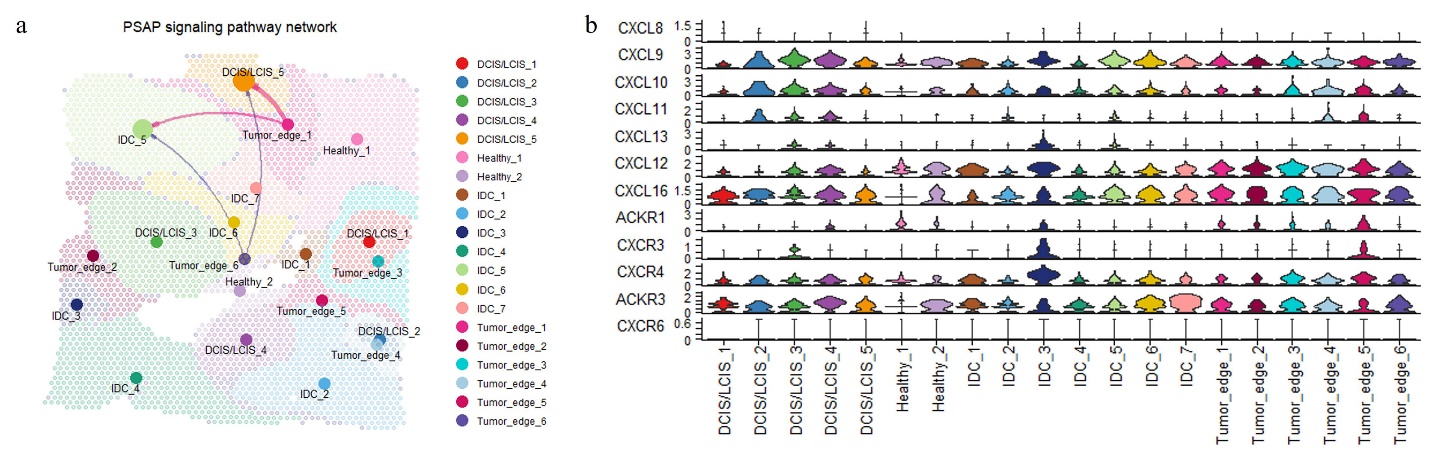


**Figure S10.** (a) Spatial map with signaling overlay of the PSAP Signaling Pathway Network for breast cancer. (b) Gene expression in CXCL Signaling Pathway for breast cancer.

**
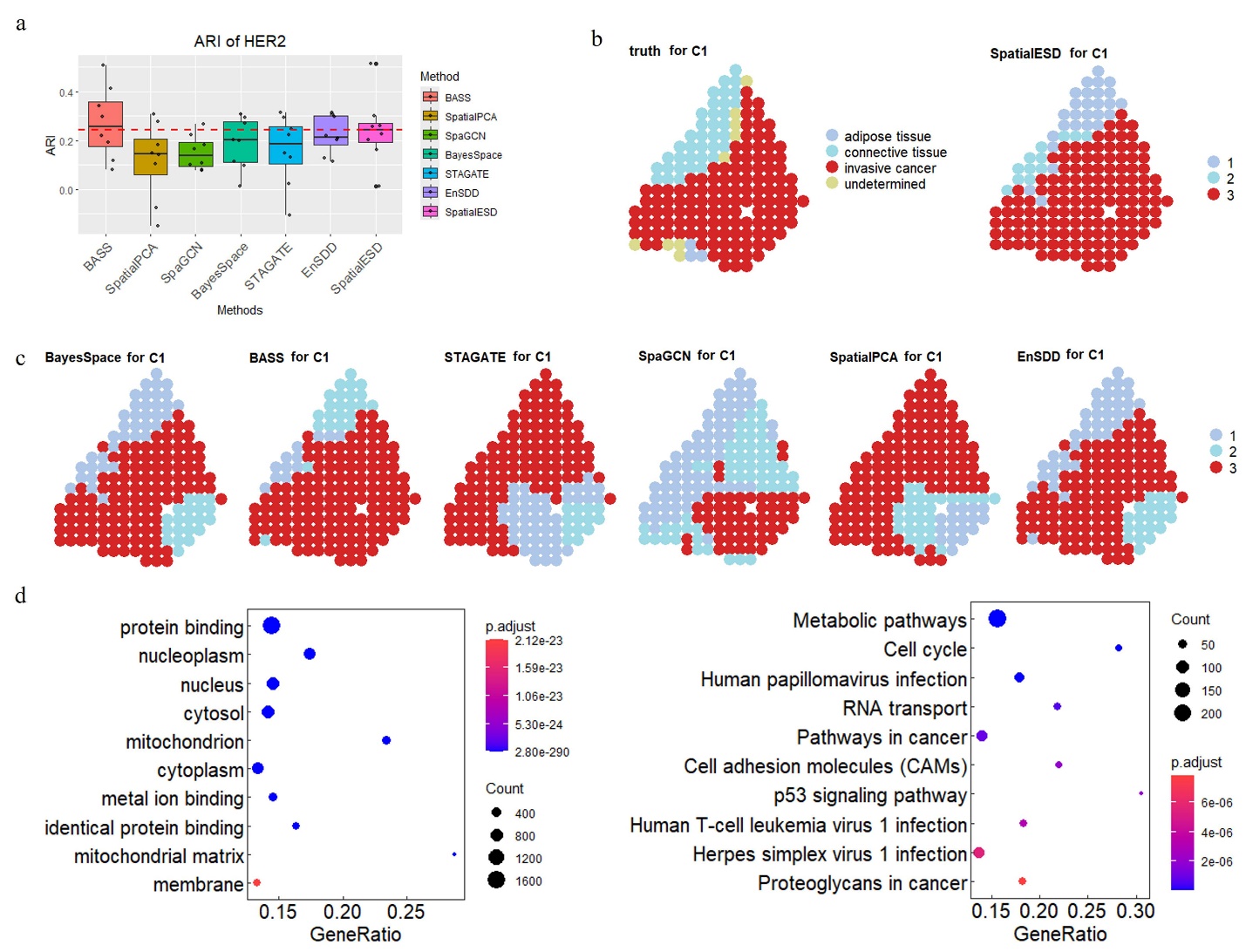
Figure S11.** SpatialESD enhances tissue structure detection in HER2. (a) Boxplots of ARI. (b-c) Visualization of spatial domains using the SpatialESD method and several SDD methods. (d) Enrichment analysis of differentially expressed genes (Left: GO enrichment; Right: KEGG pathway enrichment).


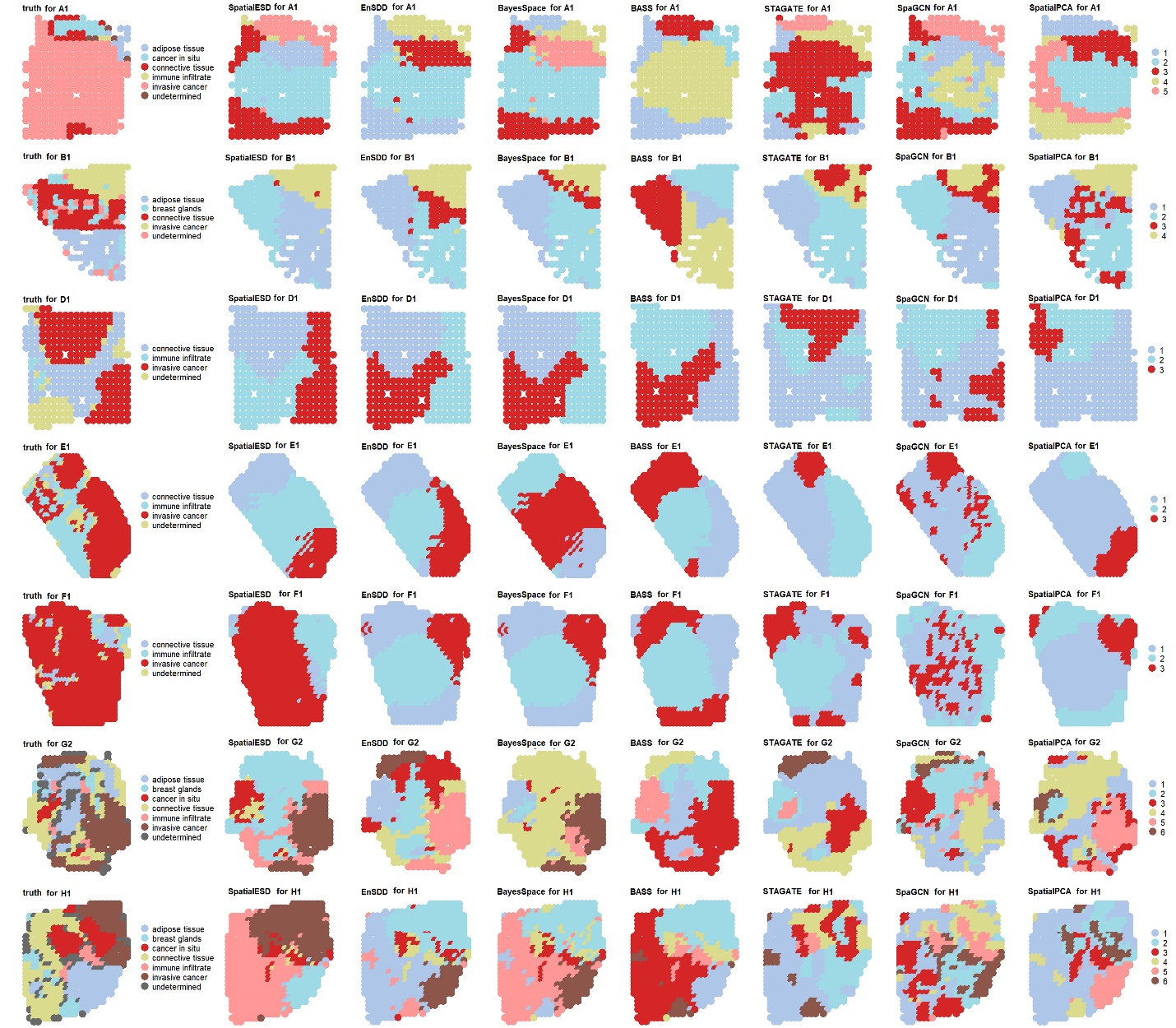


**Figure S12.** Spatial domain visualization results for different methods on 8 slices of the HER2.


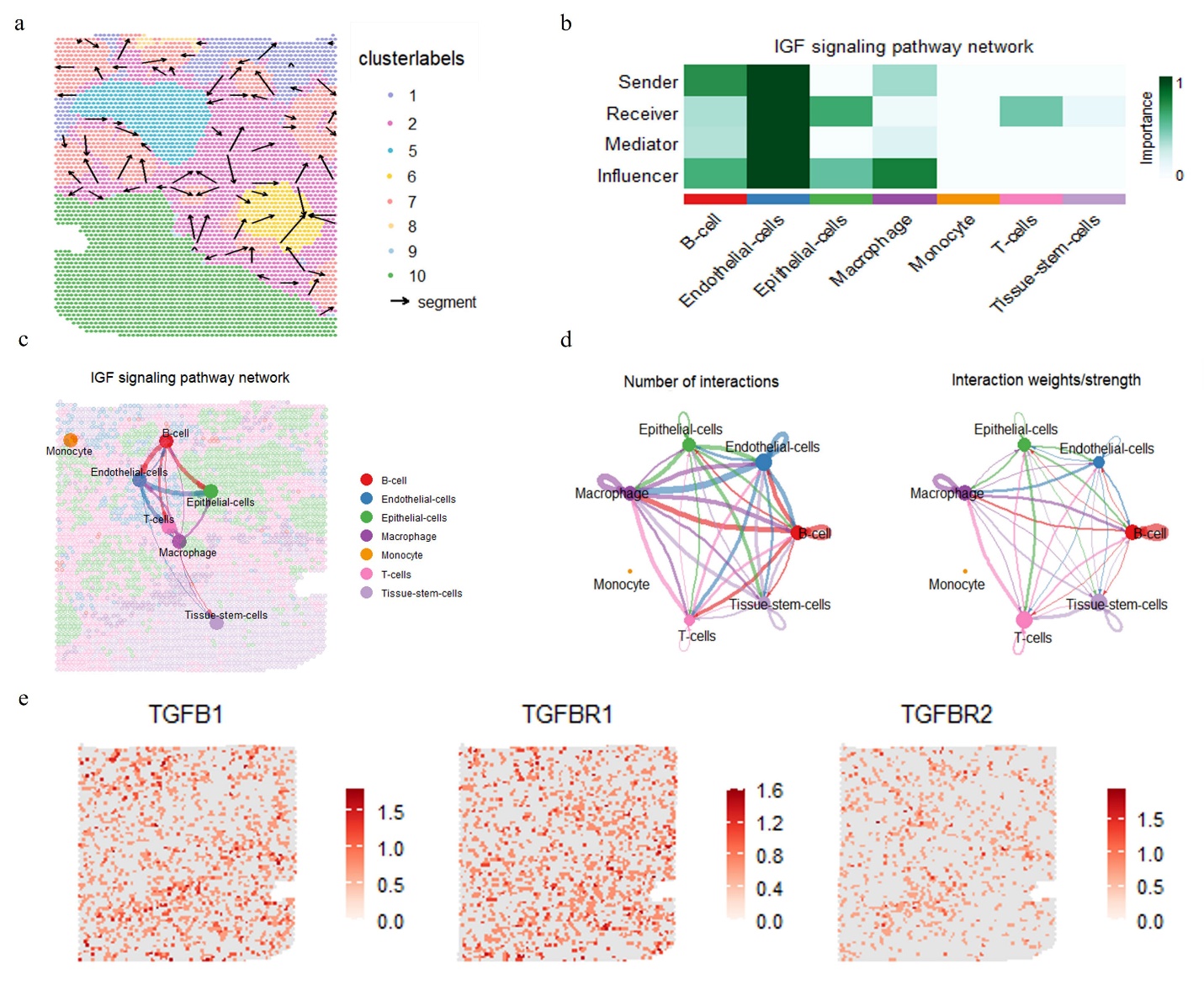


**Figure S13.** (a) Trajectory analysis for IDC. (b) Heatmap of Centrality Scores in IGF Signaling Pathway Network for IDC. (c) Spatial map with signaling overlay of the IGF Signaling Pathway Network for IDC. (d) Number of interactions and interaction weights for IDC. (e) Spatial Distribution of TGFB1, TGFBR1, and TGFBR2 Expression for IDC.


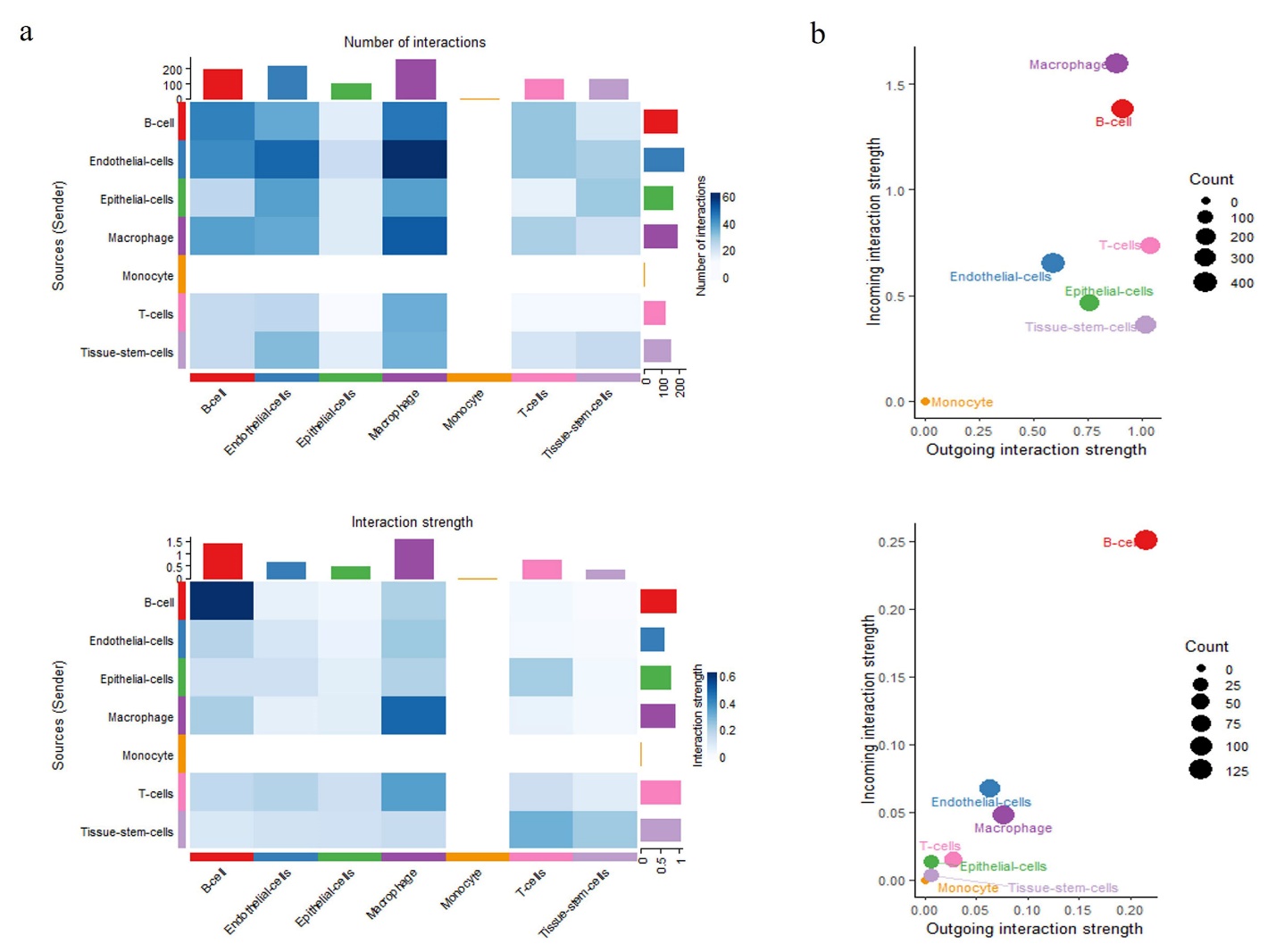


**Figure S14.** (a) Number of Interactions and Interaction Strength for IDC. (b) Global and CXCL/CCL- Specific Signaling Role Analysis in Cell-Cell Communication for IDC.


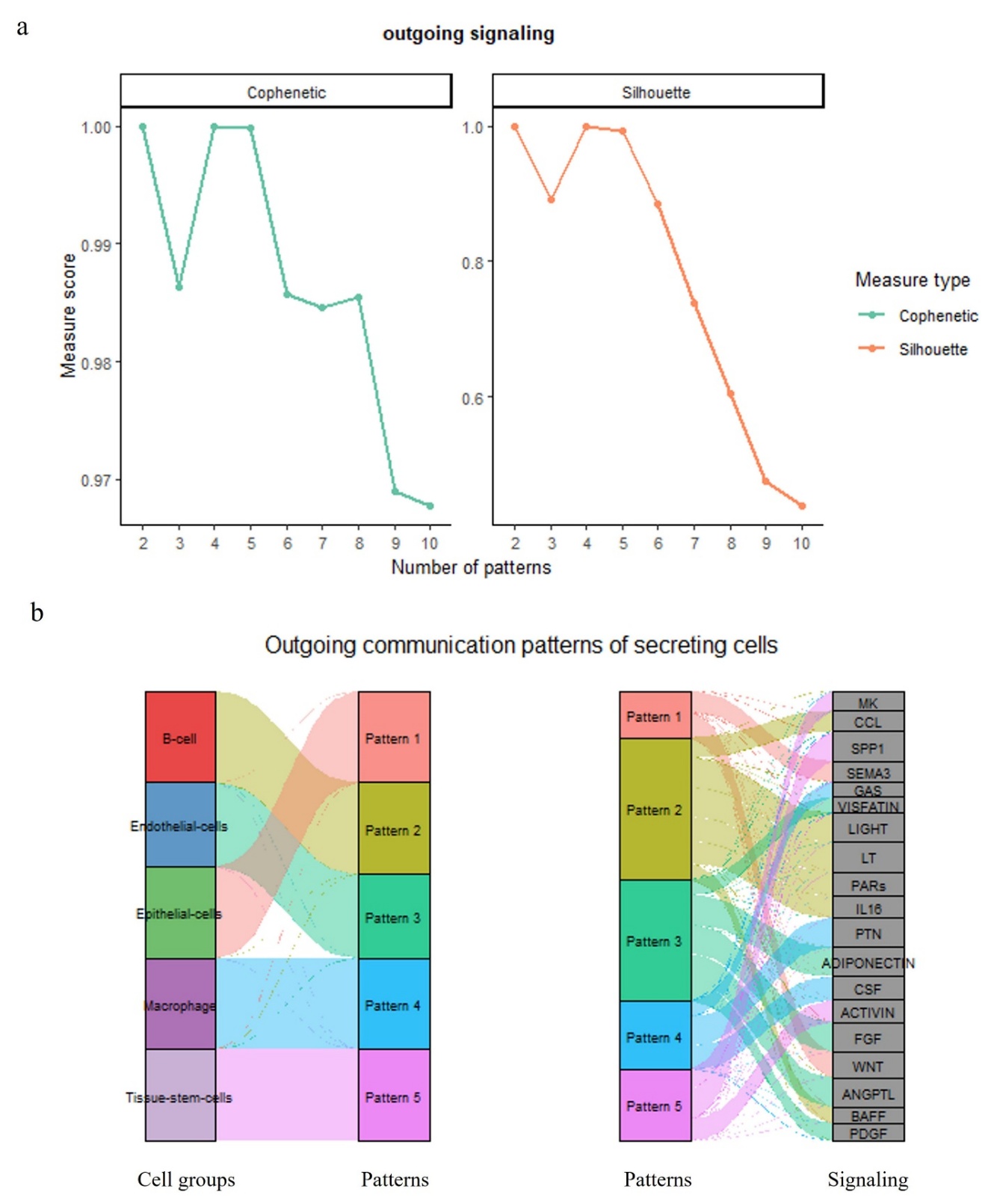


**Figure S15.** (a) Determination of the number of inferred outgoing communication patterns for IDC. (b) River plot showing outgoing communication patterns of secreting cells for IDC.


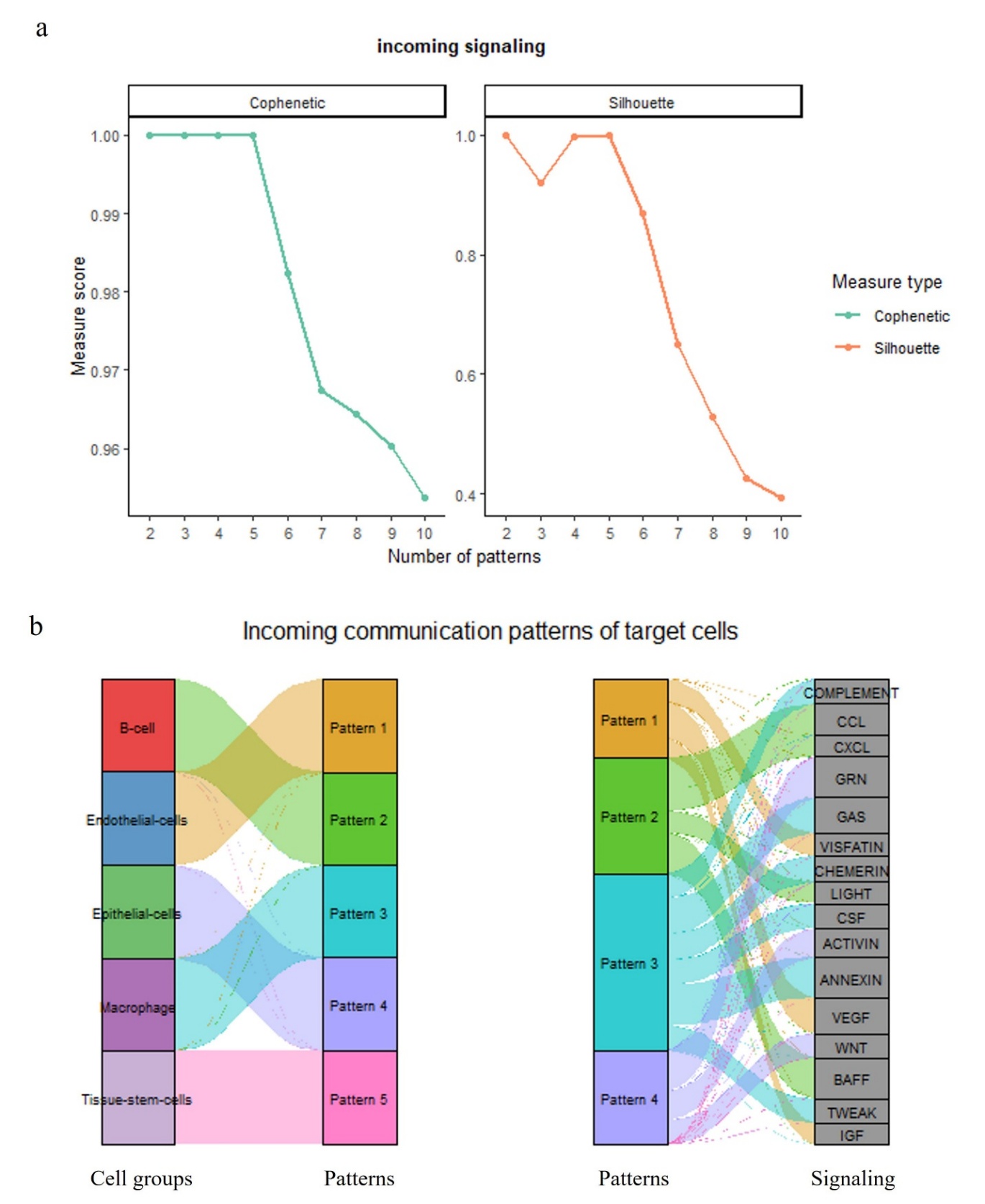


**Figure S16.** (a) Determination of the number of inferred incoming communication patterns for IDC. (b) River plot showing incoming communication patterns of secreting cells for IDC.


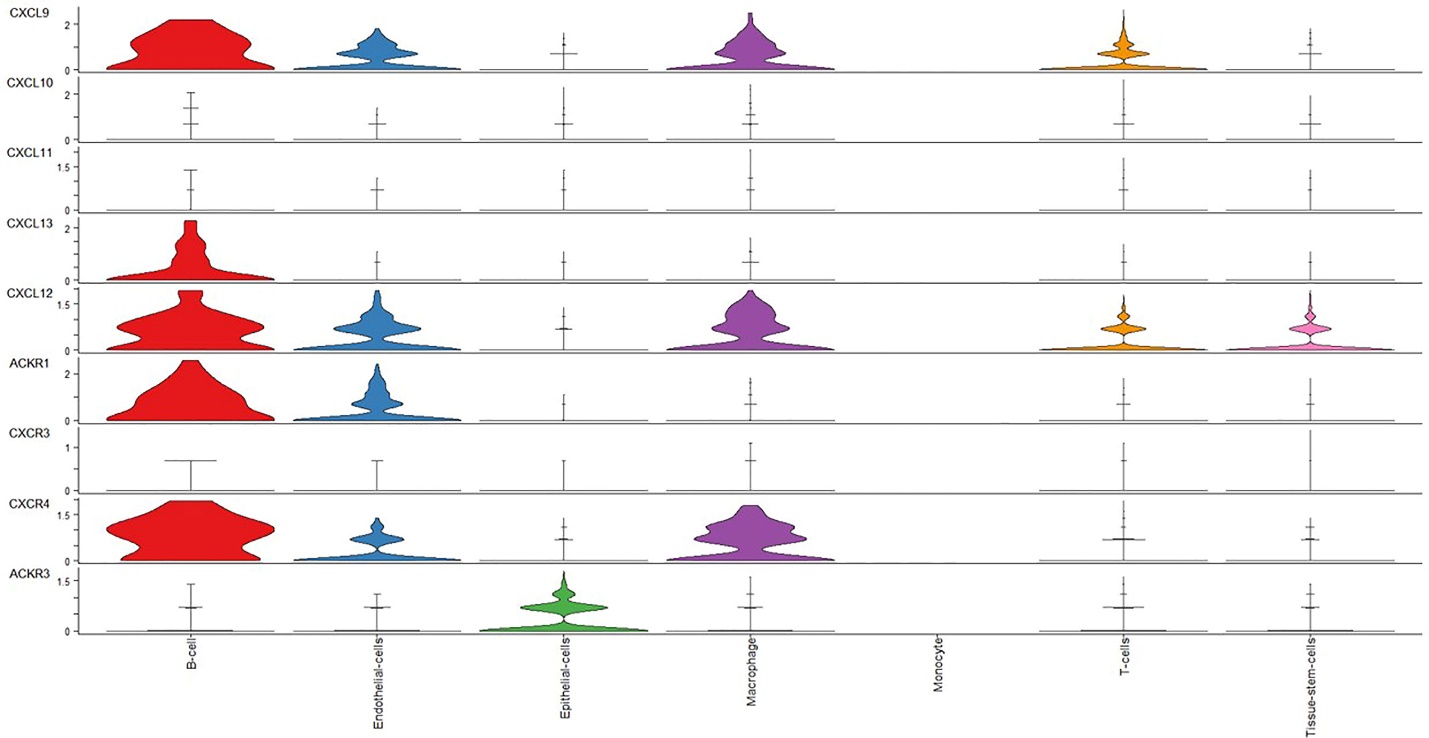


**Figure S17.** Gene Expression in CXCL Signaling Pathway for IDC.


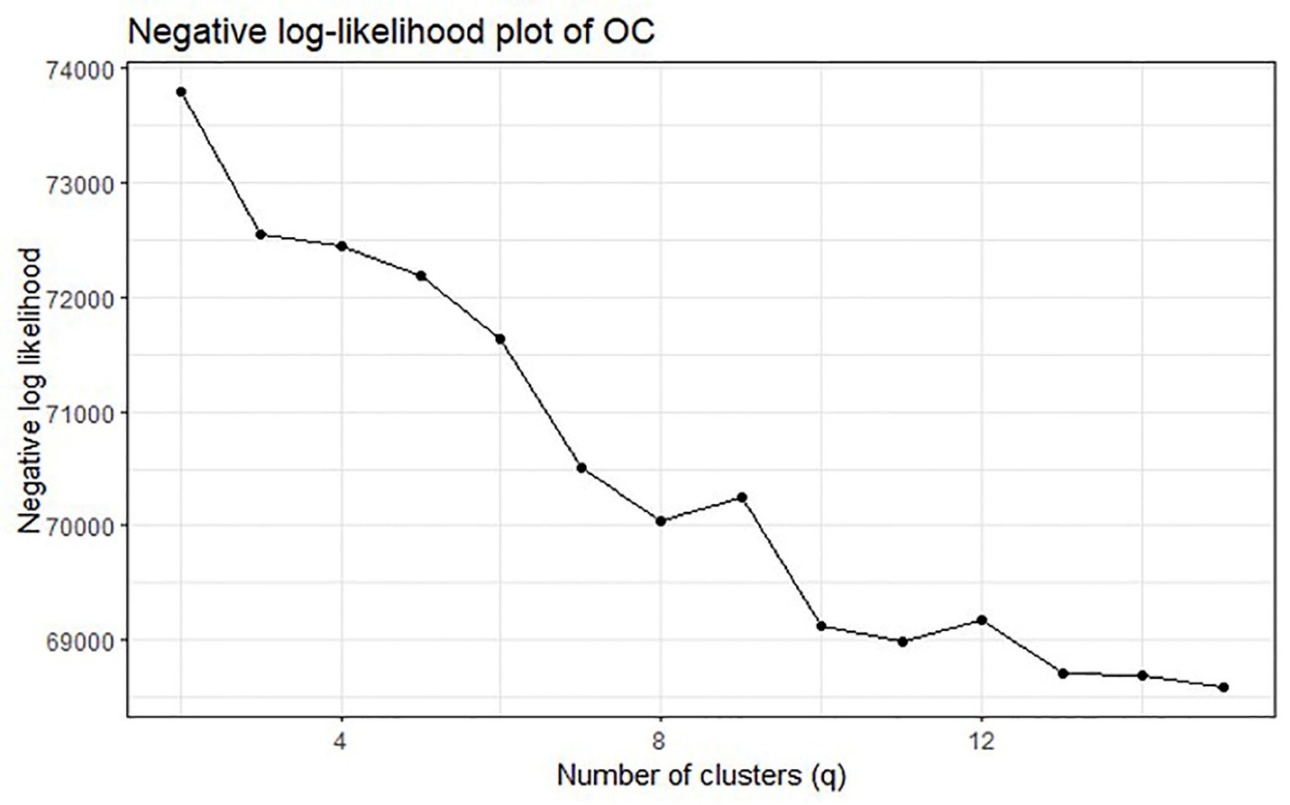


**Figure S18.** The optimal number of clusters for the OC dataset is determined to be 8 based on the elbow plot.


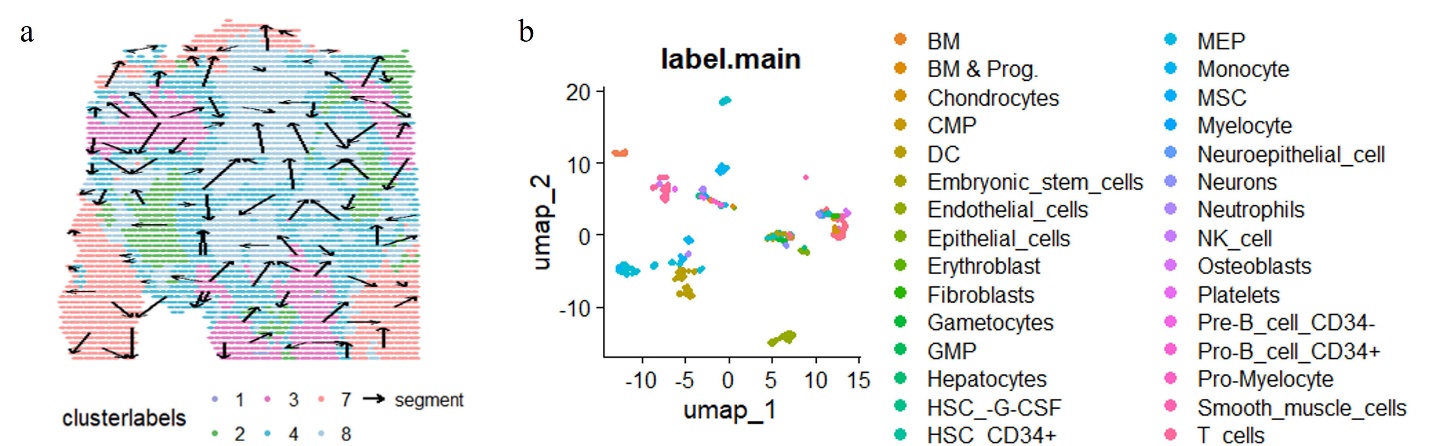


**Figure S19.** (a) Trajectory analysis for OC. (b) Integration of the OC dataset with single-cell data.


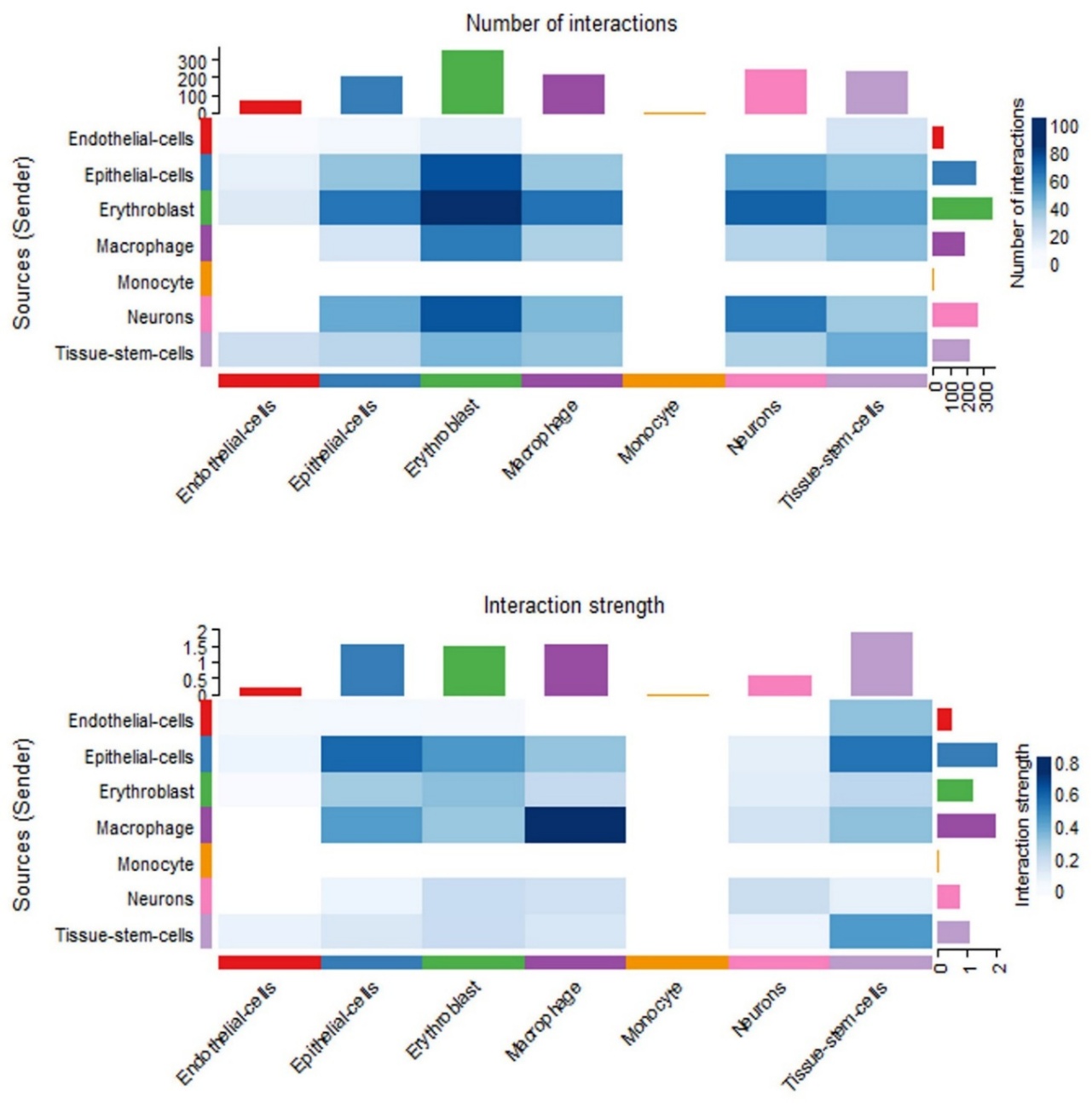


**Figure S20.** Number of Interactions and Interaction Strength for OC.


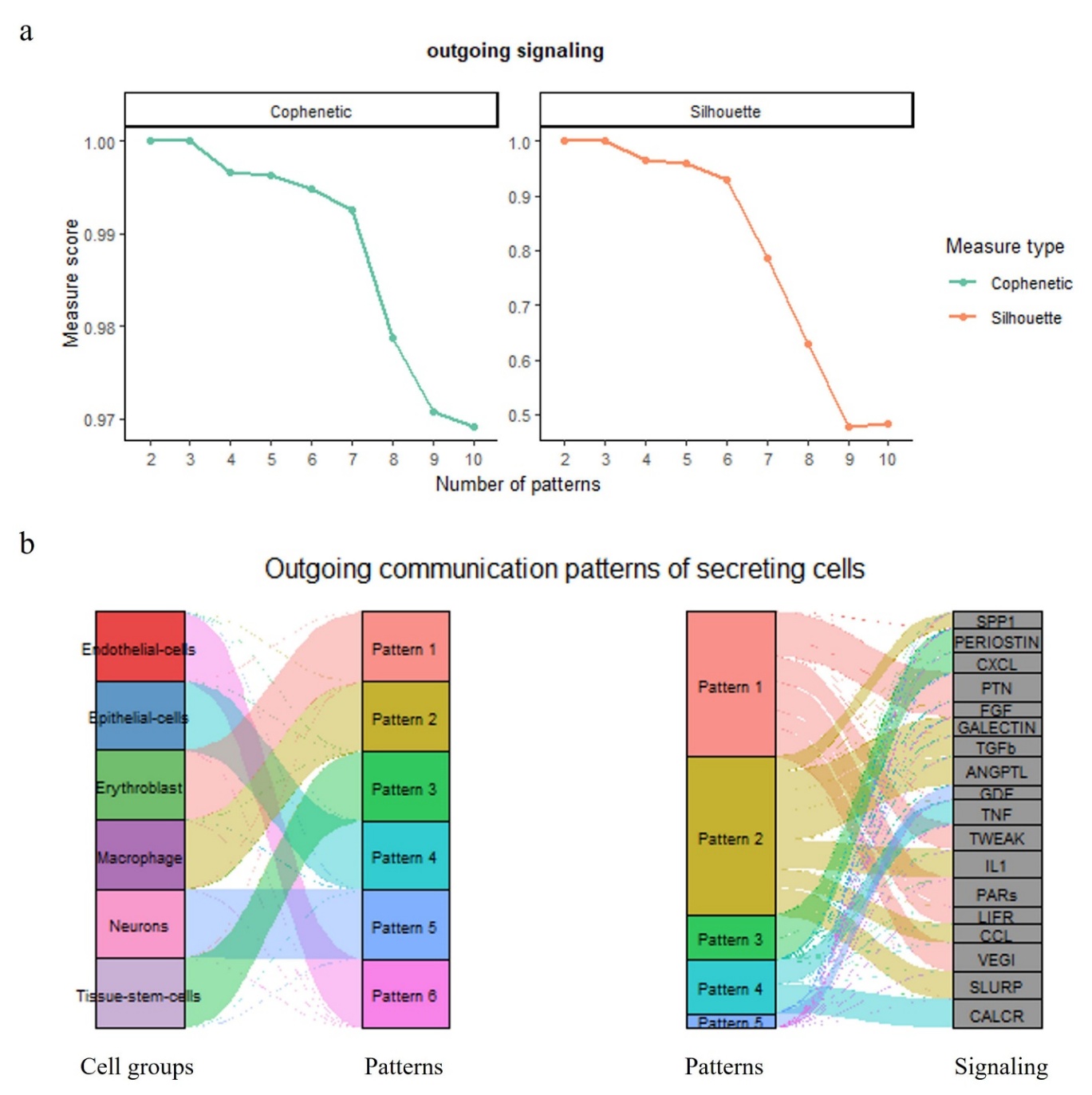


**Figure S21.** (a) Determination of the number of inferred outgoing communication patterns for OC. (b) River plot showing outgoing communication patterns of secreting cells for OC.


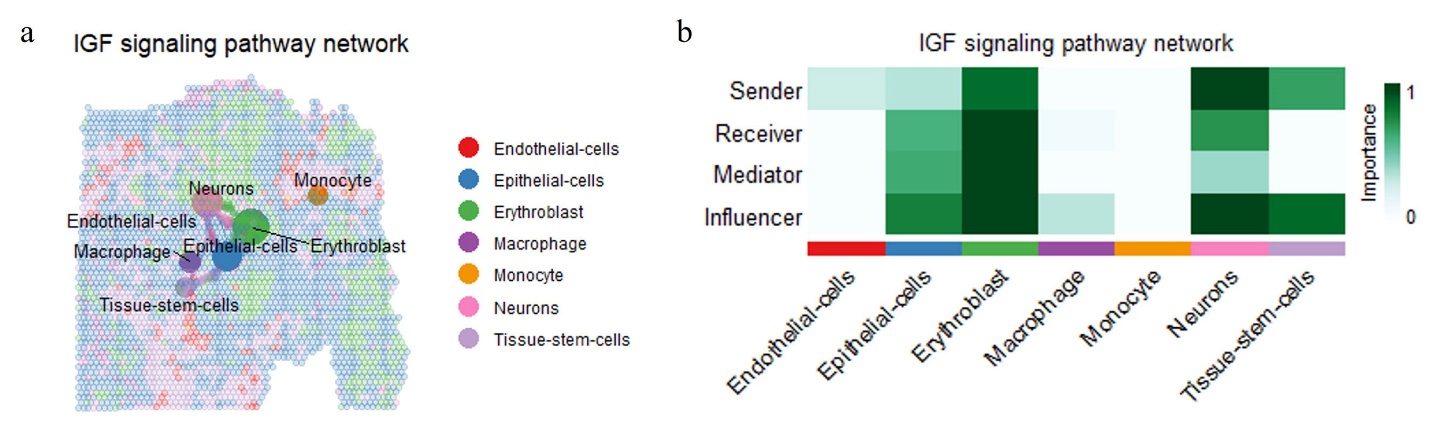


**Figure S22.** (a) Spatial map with signaling overlay of the IGF Signaling Pathway Network for OC. (b) Heatmap of Centrality Scores in IGF Signaling Pathway Network for OC.


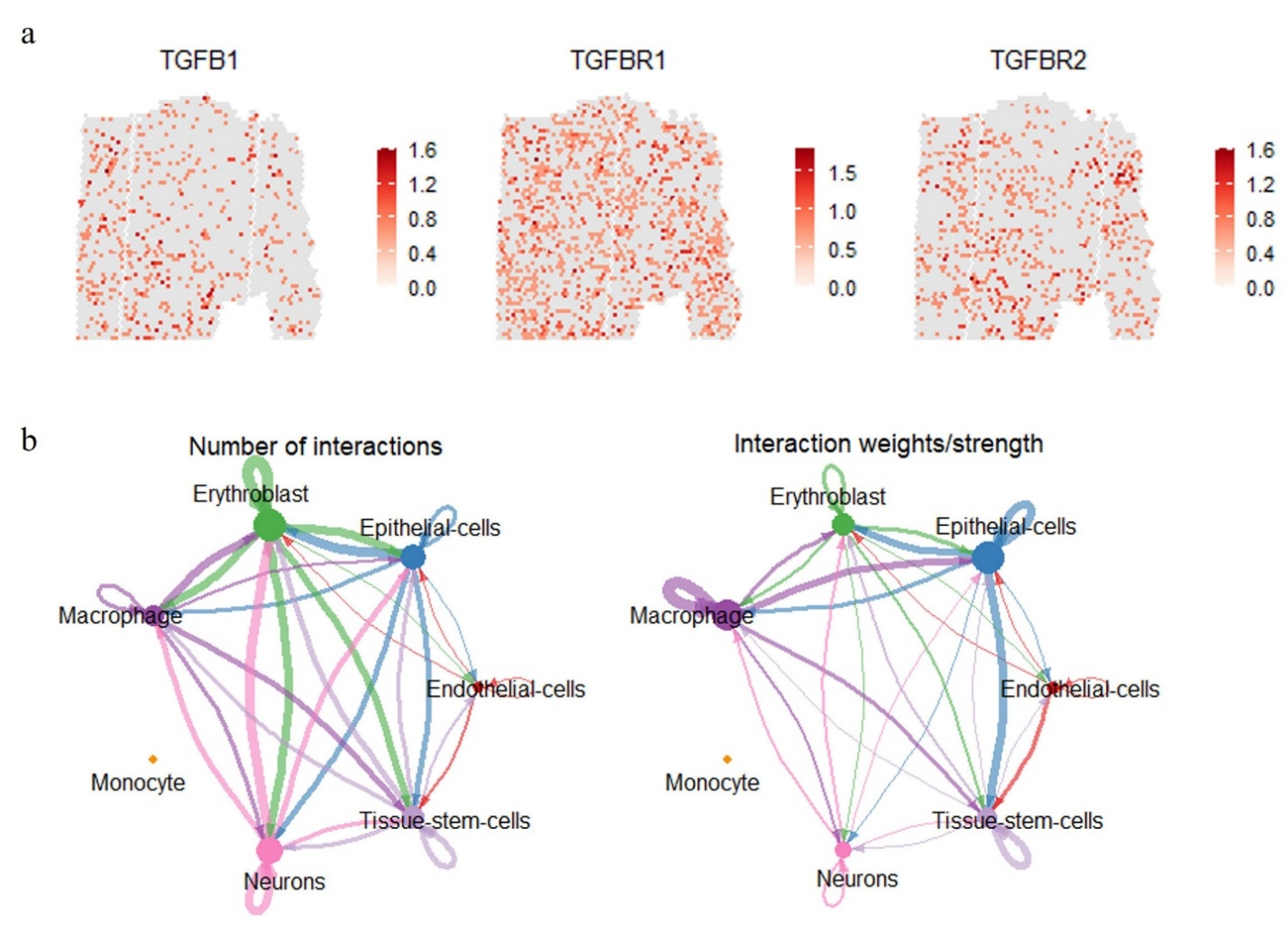


**Figure S23.** (a) Spatial Distribution of TGFB1, TGFBR1, and TGFBR2 Expression for OC. (b) Number of interactions and interaction weights for OC.


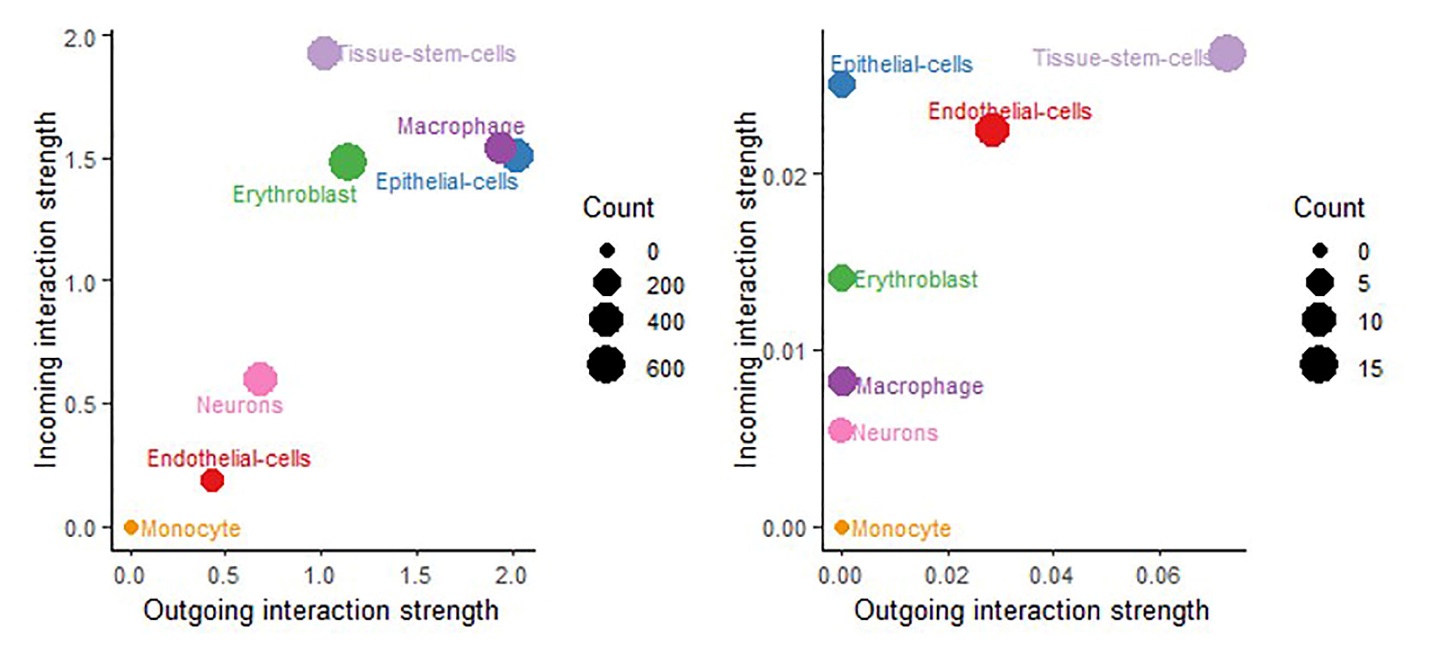


**Figure S24.** Global and CXCL/CCL- Specific Signaling Role Analysis in Cell-Cell Communication for OC.


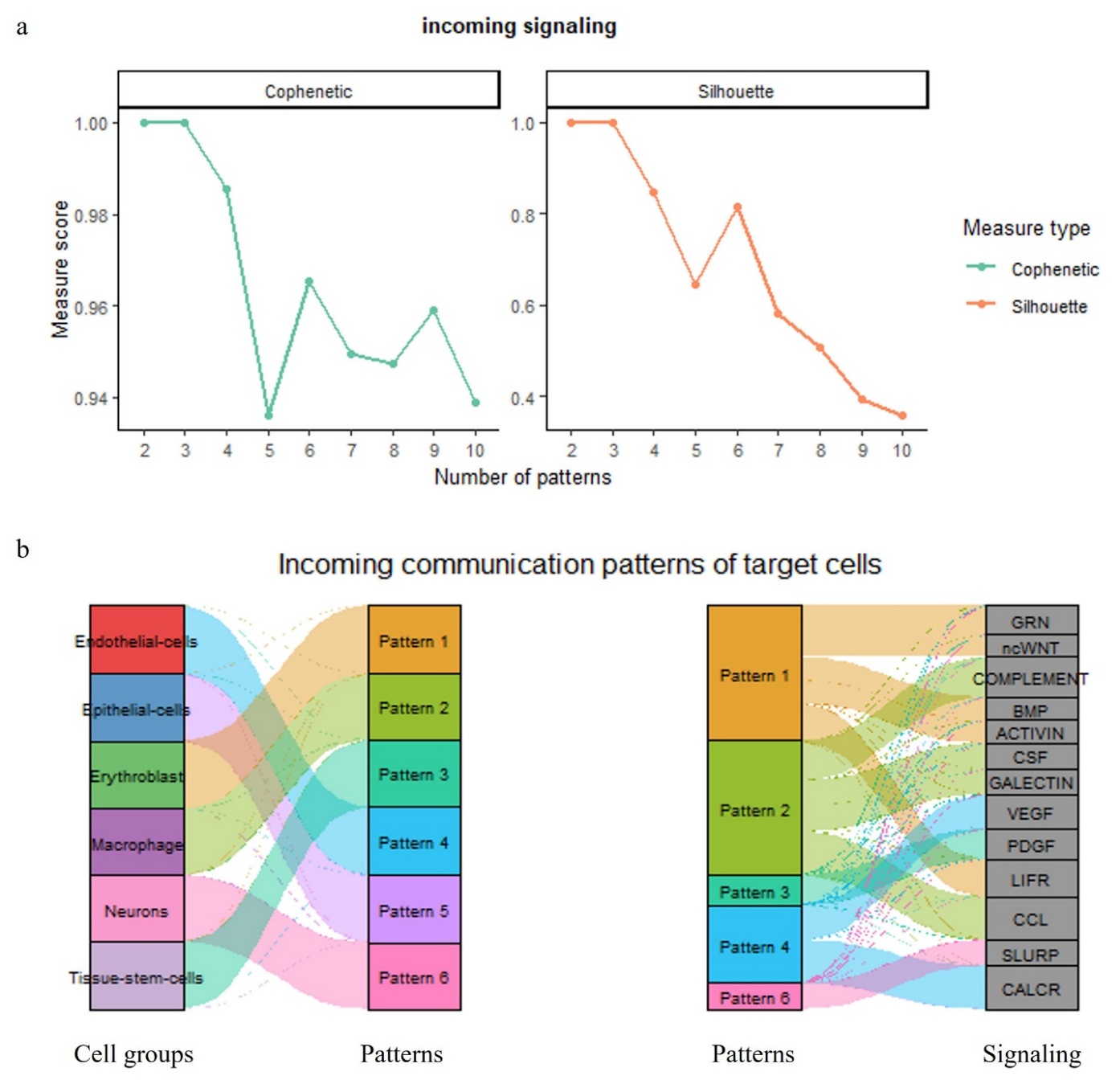


**Figure S25.** (a) Determination of the number of inferred incoming communication patterns for OC. (b) Incoming communication patterns of target cells for OC.


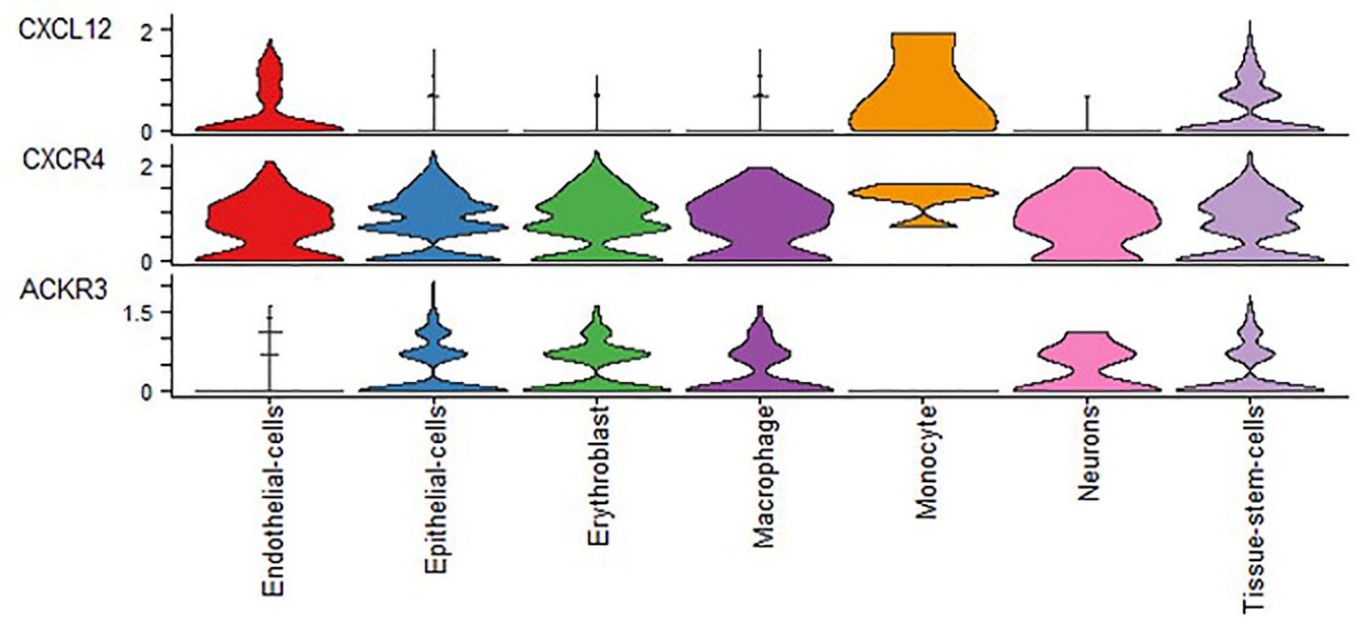


**Figure S26.** Gene Expression in CXCL Signaling Pathway for OC.


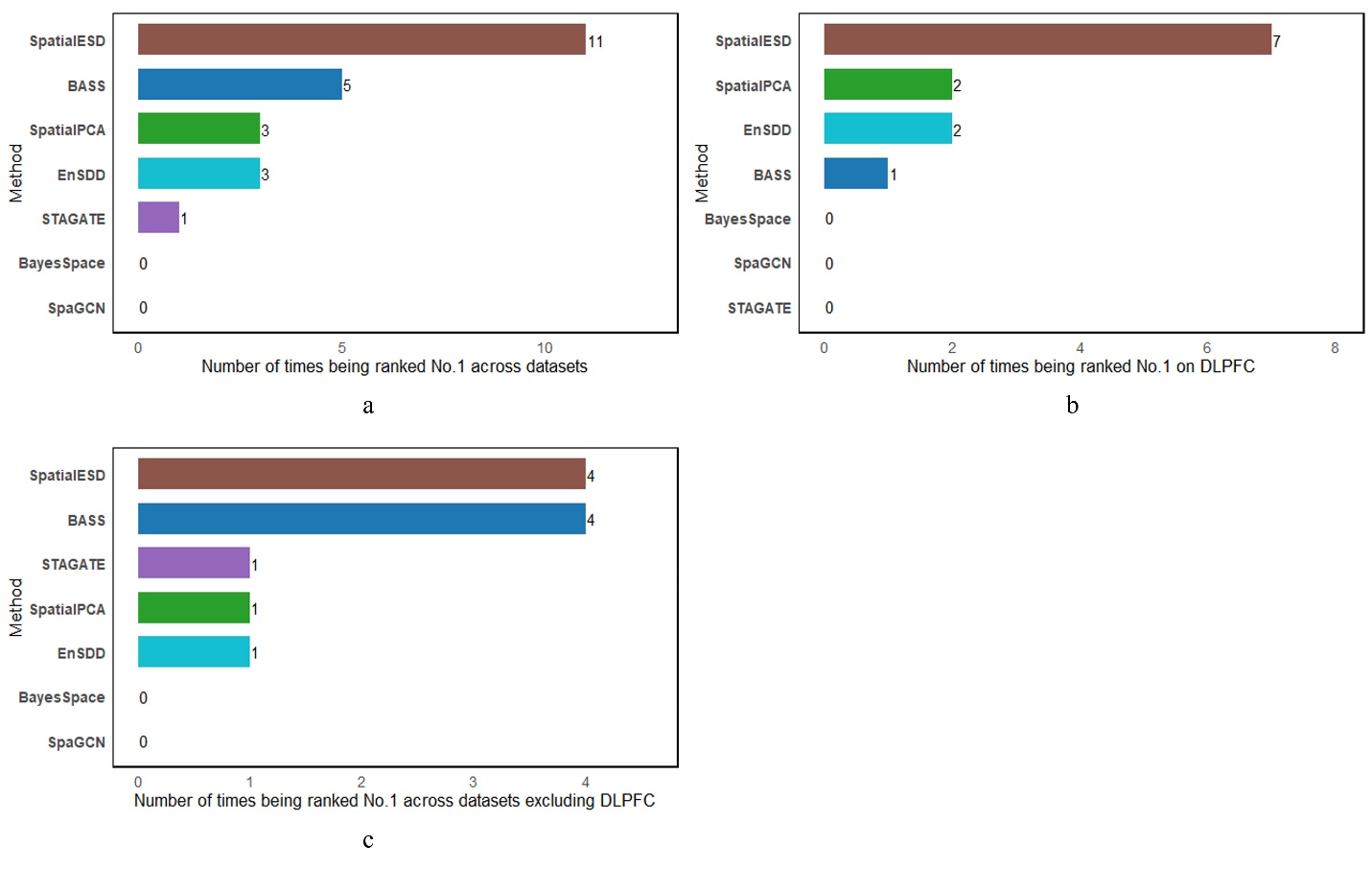


**Figure S27.** The number of times that each method is ranked in the first position. (a) all datasets. (b) DLPFC. (c) all datasets excluding DLPFC.


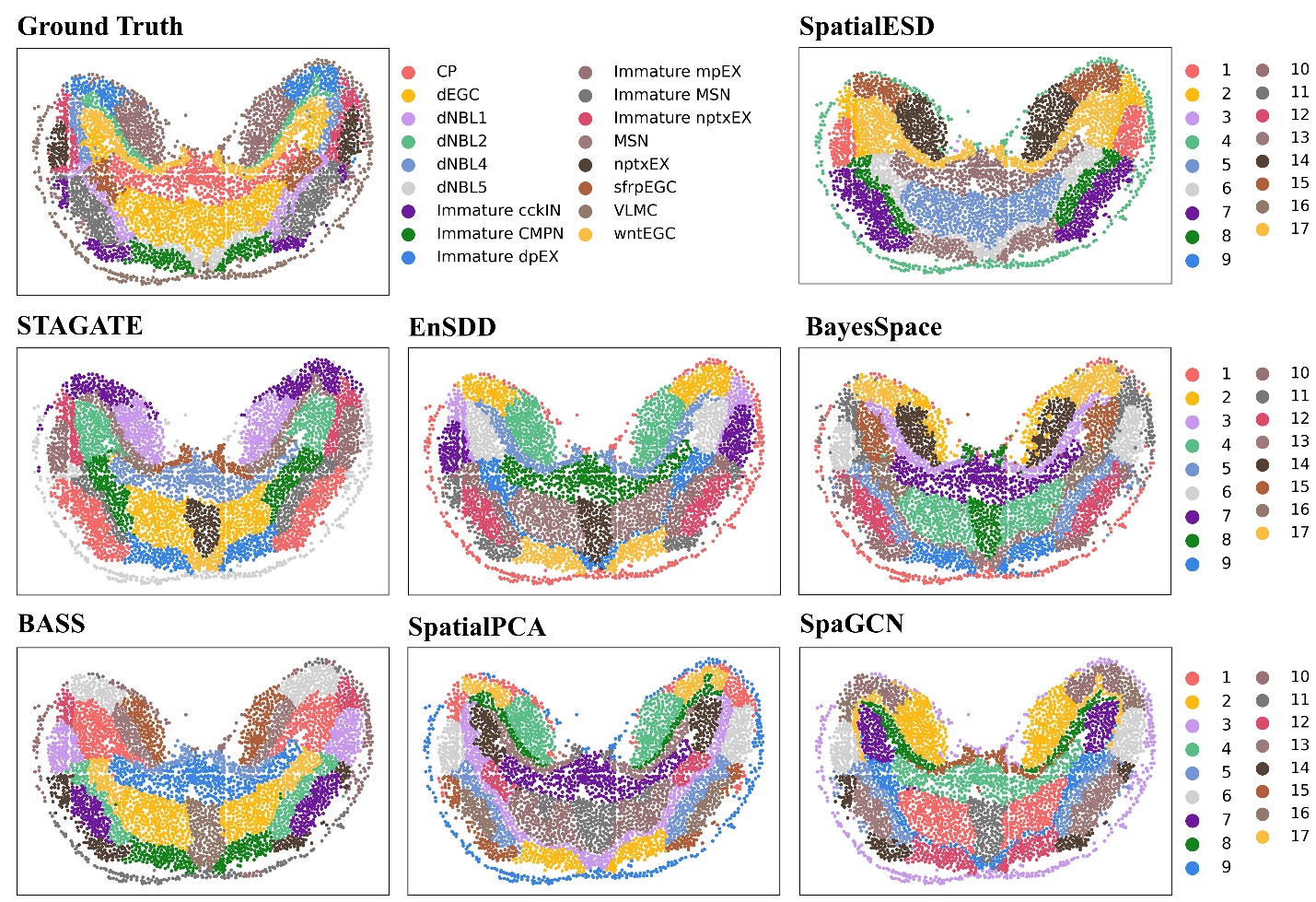


**Figure S28.** Cell cluster visualization of SpatialESD and EnSDD constructed from the same base clustering, and individual base methods on the axolotl telencephalon Stereo-seq dataset.


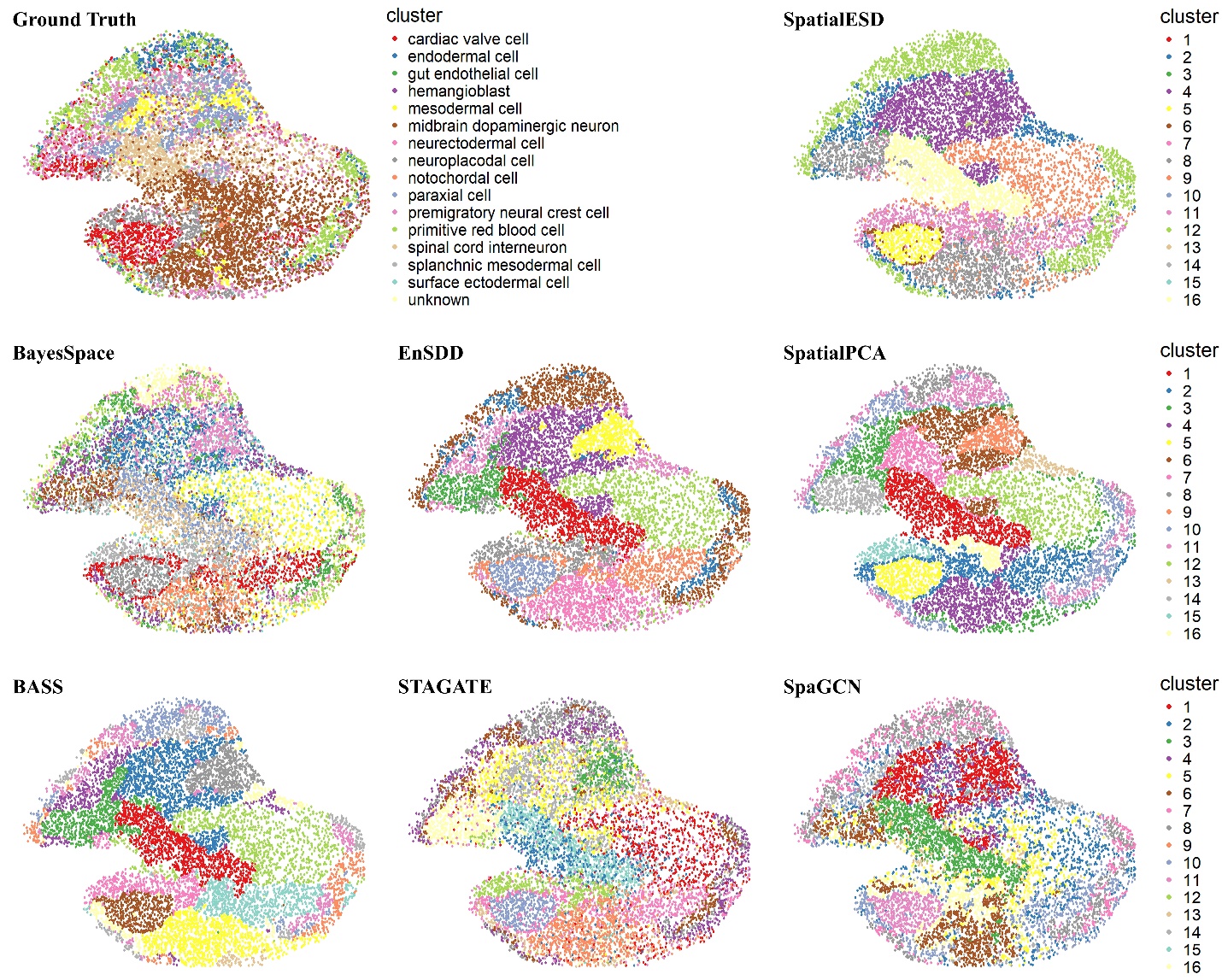


**Figure S29.** Cell cluster visualization of SpatialESD and EnSDD constructed from the same base clustering, and individual base methods on the mouse embryo Slide-seqV2 dataset.





**Figure S30.** Sensitivity analysis of random walk length across DLPFC (brain) and breast cancer (tumor) slices.

**Supplementary Tables:**

**Table S1.** ARI Results of Different SDD Methods for Simulated Data.

|  | BayesSpace | BASS | SpaGCN | STAGATE | SpatialPCA | EnSDD | SpatialESD |
| --- | --- | --- | --- | --- | --- | --- | --- |
| 01 | 0.369 | 0.227 | 0.196 | 0.433 | 0.491 | 0.402 | 0.475 |
| 02 | 0.245 | 0.213 | 0.362 | 0.462 | 0.366 | 0.314 | **0.500** |
| 03 | 0.390 | 0.322 | 0.186 | 0.478 | 0.366 | 0.329 | **0.503** |
| 04 | 0.371 | 0.288 | 0.213 | 0.362 | 0.363 | 0.321 | **0.479** |
| 05 | 0.223 | 0.203 | 0.347 | 0.444 | 0.379 | 0.357 | 0.390 |
| 06 | 0.219 | 0.300 | 0.314 | 0.444 | 0.662 | 0.393 | 0.550 |
| 07 | 0.388 | 0.233 | 0.363 | 0.220 | 0.658 | 0.319 | 0.542 |
| 08 | 0.385 | 0.210 | 0.216 | 0.433 | 0.534 | 0.267 | 0.479 |
| 09 | 0.383 | 0.216 | 0.208 | 0.456 | 0.669 | 0.387 | 0.523 |
| 10 | 0.384 | 0.210 | 0.290 | 0.360 | 0.509 | 0.340 | **0.511** |
| 11 | 0.369 | 0.210 | 0.222 | 0.369 | 0.344 | 0.404 | **0.483** |
| 12 | 0.194 | 0.231 | 0.363 | 0.425 | 0.495 | 0.323 | 0.356 |
| 13 | 0.217 | 0.217 | 0.365 | 0.202 | 0.376 | 0.308 | **0.534** |
| 14 | 0.238 | 0.227 | 0.188 | 0.314 | 0.395 | 0.285 | **0.523** |
| 15 | 0.385 | 0.205 | 0.324 | 0.285 | 0.394 | 0.323 | **0.695** |
| 16 | 0.394 | 0.228 | 0.226 | 0.430 | 0.516 | 0.261 | 0.367 |
| 17 | 0.368 | 0.211 | 0.351 | 0.439 | 0.382 | 0.288 | **0.523** |
| 18 | 0.245 | 0.347 | 0.192 | 0.362 | 0.366 | 0.323 | **0.722** |
| 19 | 0.387 | 0.228 | 0.369 | 0.365 | 0.391 | 0.410 | **0.540** |
| 20 | 0.377 | 0.222 | 0.353 | 0.442 | 0.677 | 0.414 | 0.535 |

**Table S2.** ARI Results of Different SDD Methods for DLPFC.

|  | BayesSpace | BASS | SpaGCN | STAGATE | SpatialPCA | WEST* | EnSDD | SpatialESD |
| --- | --- | --- | --- | --- | --- | --- | --- | --- |
| 151507 | 0.468 | 0.447 | 0.471 | 0.538 | 0.539 | 0.480 | 0.538 | 0.562 |
| 151508 | 0.437 | 0.406 | 0.395 | 0.456 | 0.453 | 0.450 | 0.508 | 0.317 |
| 151509 | 0.381 | 0.431 | 0.459 | 0.325 | 0.554 | 0.370 | 0.463 | 0.435 |
| 151510 | 0.374 | 0.420 | 0.430 | 0.474 | 0.419 | 0.390 | 0.474 | 0.533 |
| 151669 | 0.468 | 0.382 | 0.282 | 0.451 | 0.374 | 0.360 | 0.469 | 0.542 |
| 151670 | 0.428 | 0.378 | 0.369 | 0.439 | 0.519 | 0.360 | 0.385 | 0.69 |
| 151671 | 0.730 | 0.556 | 0.504 | 0.590 | 0.595 | 0.530 | 0.571 | 0.833 |
| 151672 | 0.426 | 0.556 | 0.557 | 0.589 | 0.527 | 0.500 | 0.583 | 0.601 |
| 151673 | 0.546 | 0.579 | 0.515 | 0.594 | 0.571 | 0.470 | 0.591 | 0.658 |
| 151674 | 0.291 | 0.637 | 0.385 | 0.557 | 0.547 | 0.450 | 0.626 | 0.596 |
| 151675 | 0.525 | 0.606 | 0.352 | 0.580 | 0.544 | 0.510 | 0.640 | 0.610 |
| 151676 | 0.364 | 0.607 | 0.328 | 0.426 | 0.629 | 0.430 | 0.488 | 0.428 |

* Indicates results reported in the original paper.

**Table S3.** ARI Results of Different SDD Methods for Breast Cancer.

|  | BayesSpace | BASS | SpaGCN | STAGATE | SpatialPCA | EnSDD | SpatialESD |
| --- | --- | --- | --- | --- | --- | --- | --- |
| Run1 | 0.500 | 0.667 | 0.542 | 0.456 | 0.319 | 0.566 | 0.636 |
| Run2 | 0.446 | 0.657 | 0.540 | 0.459 | 0.319 | 0.566 | 0.656 |
| Run3 | 0.505 | 0.664 | 0.512 | 0.556 | 0.319 | 0.578 | 0.667 |
| Run4 | 0.493 | 0.667 | 0.482 | 0.531 | 0.319 | 0.591 | 0.641 |
| Run5 | 0.455 | 0.681 | 0.500 | 0.553 | 0.319 | 0.684 | 0.620 |
| Run6 | 0.480 | 0.674 | 0.503 | 0.510 | 0.319 | 0.579 | 0.617 |
| Run7 | 0.479 | 0.671 | 0.508 | 0.554 | 0.319 | 0.474 | 0.631 |
| Run8 | 0.501 | 0.647 | 0.494 | 0.485 | 0.319 | 0.511 | 0.604 |
| Run9 | 0.494 | 0.676 | 0.509 | 0.513 | 0.319 | 0.515 | 0.617 |
| Run10 | 0.506 | 0.630 | 0.475 | 0.468 | 0.319 | 0.549 | 0.609 |

**Table S4.** ARI Results of Different SDD Methods for HER2.

|  | BayesSpace | BASS | SpaGCN | STAGATE | SpatialPCA | EnSDD | SpatialESD |
| --- | --- | --- | --- | --- | --- | --- | --- |
| A1 | 0.271 | 0.412 | 0.107 | 0.313 | 0.142 | 0.218 | 0.226 |
| B1 | 0.199 | 0.342 | 0.221 | 0.221 | 0.276 | 0.314 | 0.257 |
| C1 | 0.309 | 0.507 | 0.099 | -0.106 | -0.150 | 0.297 | **0.514** |
| D1 | 0.203 | 0.193 | 0.182 | 0.130 | 0.103 | 0.207 | **0.203** |
| E1 | 0.011 | 0.119 | 0.081 | 0.246 | -0.077 | 0.126 | 0.011 |
| F1 | 0.115 | 0.079 | 0.078 | 0.022 | 0.147 | 0.115 | **0.300** |
| G2 | 0.098 | 0.220 | 0.165 | 0.147 | 0.182 | 0.201 | 0.160 |
| H1 | 0.292 | 0.296 | 0.267 | 0.293 | 0.308 | 0.306 | 0.259 |

**Table S5.** LISI Results of Different SDD Methods for IDC (Number of Clusters from 2 to 10).

| Cluster | BayesSpace | BASS | SpaGCN | STAGATE | SpatialPCA | EnSDD | SpatialESD |
| --- | --- | --- | --- | --- | --- | --- | --- |
| 2 | 1.000 | 1.166 | 1.000 | 1.000 | 1.000 | 1.000 | 1.000 |
| 3 | 1.000 | 1.063 | 1.059 | 1.000 | 1.000 | 1.000 | 1.000 |
| 4 | 1.065 | 1.160 | 1.115 | 1.008 | 1.002 | 1.076 | 1.000 |
| 5 | 1.158 | 1.199 | 1.129 | 1.032 | 1.025 | 1.088 | 1.000 |
| 6 | 1.098 | 1.281 | 1.181 | 1.168 | 1.082 | 1.076 | 1.002 |
| 7 | 1.204 | 1.324 | 1.183 | 1.183 | 1.298 | 1.208 | 1.062 |
| 8 | 1.200 | 1.400 | 1.388 | 1.260 | 1.492 | 1.298 | 1.041 |
| 9 | 1.367 | 1.394 | 1.418 | 1.274 | 1.510 | 1.298 | 1.099 |
| 10 | 1.447 | 1.432 | 1.386 | 1.317 | 1.515 | 1.386 | 1.170 |

**Table S6.** PAS Results of Different SDD Methods for IDC (Number of Clusters from 2 to 10).

| Cluster | BayesSpace | BASS | SpaGCN | STAGATE | SpatialPCA | EnSDD | SpatialESD |
| --- | --- | --- | --- | --- | --- | --- | --- |
| 2 | 0.002 | 0.048 | 0.016 | 0.003 | 0.006 | 0.003 | 0.003 |
| 3 | 0.008 | 0.033 | 0.029 | 0.005 | 0.009 | 0.007 | 0.006 |
| 4 | 0.027 | 0.066 | 0.049 | 0.011 | 0.011 | 0.030 | 0.008 |
| 5 | 0.040 | 0.067 | 0.050 | 0.015 | 0.017 | 0.045 | 0.016 |
| 6 | 0.041 | 0.088 | 0.071 | 0.035 | 0.028 | 0.029 | 0.017 |
| 7 | 0.055 | 0.094 | 0.074 | 0.048 | 0.058 | 0.094 | 0.037 |
| 8 | 0.058 | 0.099 | 0.110 | 0.065 | 0.112 | 0.094 | 0.032 |
| 9 | 0.077 | 0.097 | 0.119 | 0.063 | 0.113 | 0.08 | 0.041 |
| 10 | 0.111 | 0.110 | 0.112 | 0.072 | 0.115 | 0.092 | 0.073 |

**Table S7.** LISI Results of Different SDD Methods for IDC under 10 Random Seeds.

| Seed | BayesSpace | BASS | SpaGCN | STAGATE | SpatialPCA | EnSDD | SpatialESD |
| --- | --- | --- | --- | --- | --- | --- | --- |
| 1 | 1.453 | 1.432 | 1.479 | 1.307 | 1.498 | 1.316 | 1.133 |
| 2 | 1.416 | 1.437 | 1.517 | 1.325 | 1.498 | 1.286 | 1.169 |
| 3 | 1.425 | 1.37 | 1.443 | 1.386 | 1.498 | 1.293 | 1.204 |
| 4 | 1.394 | 1.433 | 1.303 | 1.290 | 1.498 | 1.298 | 1.147 |
| 5 | 1.419 | 1.373 | 1.412 | 1.278 | 1.498 | 1.269 | 1.162 |
| 6 | 1.446 | 1.442 | 1.459 | 1.311 | 1.498 | 1.302 | 1.189 |
| 7 | 1.383 | 1.441 | 1.444 | 1.274 | 1.498 | 1.313 | 1.042 |
| 8 | 1.383 | 1.427 | 1.465 | 1.277 | 1.498 | 1.282 | 1.179 |
| 9 | 1.392 | 1.434 | 1.487 | 1.437 | 1.498 | 1.351 | 1.306 |
| 10 | 1.419 | 1.353 | 1.334 | 1.324 | 1.498 | 1.29 | 1.184 |

**Table S8.** PAS Results of Different SDD Methods for IDC under 10 Random Seeds.

| Seed | BayesSpace | BASS | SpaGCN | STAGATE | SpatialPCA | EnSDD | SpatialESD |
| --- | --- | --- | --- | --- | --- | --- | --- |
| 1 | 0.109 | 0.11 | 0.119 | 0.074 | 0.094 | 0.085 | 0.058 |
| 2 | 0.092 | 0.111 | 0.125 | 0.078 | 0.094 | 0.076 | 0.077 |
| 3 | 0.084 | 0.095 | 0.117 | 0.082 | 0.094 | 0.074 | 0.056 |
| 4 | 0.100 | 0.112 | 0.099 | 0.064 | 0.094 | 0.094 | 0.066 |
| 5 | 0.092 | 0.100 | 0.115 | 0.066 | 0.094 | 0.072 | 0.080 |
| 6 | 0.099 | 0.114 | 0.102 | 0.073 | 0.094 | 0.084 | 0.050 |
| 7 | 0.08 | 0.110 | 0.135 | 0.067 | 0.094 | 0.098 | 0.029 |
| 8 | 0.081 | 0.112 | 0.123 | 0.076 | 0.094 | 0.082 | 0.054 |
| 9 | 0.099 | 0.113 | 0.135 | 0.104 | 0.094 | 0.117 | 0.086 |
| 10 | 0.092 | 0.107 | 0.078 | 0.072 | 0.094 | 0.079 | 0.048 |

**Table S9.** LISI Results of Different SDD Methods for OC (Number of Clusters from 2 to 10).

| Cluster | BayesSpace | BASS | SpaGCN | STAGATE | SpatialPCA | EnSDD | SpatialESD |
| --- | --- | --- | --- | --- | --- | --- | --- |
| 2 | 1.021 | 1.172 | 1.168 | 1.043 | 1.020 | 1.119 | 1.149 |
| 3 | 1.101 | 1.232 | 1.512 | 1.251 | 1.379 | 1.175 | 1.237 |
| 4 | 1.307 | 1.498 | 1.516 | 1.415 | 1.460 | 1.458 | 1.326 |
| 5 | 1.637 | 1.763 | 1.793 | 1.463 | 1.601 | 1.619 | 1.336 |
| 6 | 1.804 | 1.829 | 1.745 | 1.600 | 1.774 | 1.735 | 1.307 |
| 7 | 1.836 | 1.921 | 1.892 | 1.935 | 1.819 | 1.748 | 1.673 |
| 8 | 1.863 | 1.993 | 1.814 | 1.982 | 1.997 | 1.895 | 1.419 |
| 9 | 1.979 | 1.980 | 2.108 | 1.76 | 2.027 | 1.869 | 1.414 |
| 10 | 2.011 | 1.969 | 2.116 | 2.01 | 2.163 | 1.926 | 1.421 |

**Table S10.** PAS Results of Different SDD Methods for OC (Number of Clusters from 2 to 10).

| Cluster | BayesSpace | BASS | SpaGCN | STAGATE | SpatialPCA | EnSDD | SpatialESD |
| --- | --- | --- | --- | --- | --- | --- | --- |
| 2 | 0.022 | 0.068 | 0.061 | 0.035 | 0.030 | 0.055 | 0.064 |
| 3 | 0.038 | 0.065 | 0.115 | 0.072 | 0.094 | 0.090 | 0.099 |
| 4 | 0.062 | 0.118 | 0.139 | 0.093 | 0.112 | 0.100 | 0.075 |
| 5 | 0.123 | 0.177 | 0.185 | 0.079 | 0.148 | 0.127 | 0.077 |
| 6 | 0.176 | 0.190 | 0.188 | 0.123 | 0.186 | 0.162 | 0.066 |
| 7 | 0.190 | 0.206 | 0.212 | 0.220 | 0.193 | 0.170 | 0.148 |
| 8 | 0.191 | 0.223 | 0.213 | 0.224 | 0.231 | 0.210 | 0.121 |
| 9 | 0.229 | 0.220 | 0.271 | 0.169 | 0.242 | 0.193 | 0.121 |
| 10 | 0.236 | 0.214 | 0.273 | 0.249 | 0.302 | 0.216 | 0.127 |

**Table S11.** LISI Results of Different SDD Methods for OC under 10 Random Seeds.

| Seed | BayesSpace | BASS | SpaGCN | STAGATE | SpatialPCA | EnSDD | SpatialESD |
| --- | --- | --- | --- | --- | --- | --- | --- |
| 1 | 1.982 | 1.820 | 1.862 | 1.996 | 1.945 | 1.856 | 1.673 |
| 2 | 1.879 | 1.766 | 2.036 | 1.773 | 1.945 | 1.794 | 1.590 |
| 3 | 1.977 | 1.780 | 2.018 | 1.830 | 1.945 | 1.799 | 1.651 |
| 4 | 1.863 | 1.763 | 1.942 | 1.882 | 1.945 | 1.817 | 1.573 |
| 5 | 1.935 | 1.799 | 2.047 | 1.965 | 1.945 | 1.877 | 1.543 |
| 6 | 1.982 | 1.843 | 1.931 | 2.126 | 1.945 | 1.889 | 1.637 |
| 7 | 1.937 | 1.824 | 2.020 | 1.910 | 1.945 | 1.786 | 1.621 |
| 8 | 1.935 | 1.809 | 1.984 | 1.967 | 1.945 | 1.831 | 1.638 |
| 9 | 1.984 | 1.773 | 1.945 | 2.101 | 1.945 | 1.855 | 1.289 |
| 10 | 1.879 | 1.79 | 2.083 | 1.821 | 1.945 | 1.813 | 1.563 |

**Table S12.** PAS Results of Different SDD Methods for OC under 10 Random Seeds.

| Seed | BayesSpace | BASS | SpaGCN | STAGATE | SpatialPCA | EnSDD | SpatialESD |
| --- | --- | --- | --- | --- | --- | --- | --- |
| 1 | 0.233 | 0.182 | 0.214 | 0.219 | 0.212 | 0.195 | 0.140 |
| 2 | 0.199 | 0.178 | 0.253 | 0.160 | 0.212 | 0.182 | 0.129 |
| 3 | 0.233 | 0.184 | 0.280 | 0.176 | 0.212 | 0.180 | 0.137 |
| 4 | 0.191 | 0.176 | 0.237 | 0.198 | 0.212 | 0.190 | 0.113 |
| 5 | 0.202 | 0.178 | 0.266 | 0.232 | 0.212 | 0.198 | 0.115 |
| 6 | 0.235 | 0.200 | 0.231 | 0.248 | 0.212 | 0.200 | 0.132 |
| 7 | 0.204 | 0.180 | 0.261 | 0.188 | 0.212 | 0.184 | 0.132 |
| 8 | 0.203 | 0.187 | 0.238 | 0.224 | 0.212 | 0.196 | 0.131 |
| 9 | 0.233 | 0.167 | 0.226 | 0.269 | 0.212 | 0.194 | 0.075 |
| 10 | 0.195 | 0.188 | 0.261 | 0.175 | 0.212 | 0.190 | 0.115 |

**Table S13.** ARI Results of Different SDD Methods on Slide-seqV2 and Stereo-seq Platforms.

|  | BayesSpace | BASS | SpaGCN | STAGATE | SpatialPCA | EnSDD | SpatialESD |
| --- | --- | --- | --- | --- | --- | --- | --- |
| Stereo-seq | 0.610 | 0.575 | 0.659 | 0.557 | 0.597 | 0.665 | **0.678** |
| slide-seq | 0.171 | 0.210 | 0.161 | 0.193 | 0.171 | 0.199 | 0.196 |

**Table S14.** Runtime of the SpatialESD Ensemble Step Compared to Base Methods.

| Dataset | Spots | BayesSpace (hours) | BASS (hours) | SpaGCN (hours) | STAGATE  (hours) | SpatialPCA (hours) | SpatialESD ensemble(seconds) |
| --- | --- | --- | --- | --- | --- | --- | --- |
| DLPFC | 3,460 to 4,789 | 0.3 | 0.2 | 0.1 | 0.1 | 0.8 | 1.0 |
| Breast cancer | 3,798 | 0.3 | 0.2 | 0.1 | 0.1 | 0.8 | 1.0 |
| HER2 | 176 to 691 | 0.01 | 0.01 | 0.1 | 0.1 | 0.1 | 0.1 |
| OC | 3,493 | 0.3 | 0.2 | 0.1 | 0.1 | 0.8 | 1.0 |
| IDC | 4,727 | 0.3 | 0.2 | 0.1 | 0.1 | 1.0 | 1.0 |

**Table S15.** Summary of Key Spatial Domain Detection Methods.

| Methods | Keyword descriptions | Histology | Website | Language |
| --- | --- | --- | --- | --- |
| BayesSpace | Bayesian model with a Markov random field | × | https://www.bioconductor.org/packages/release/bioc/html/BayesSpace | R |
| BASS | multi‑scale and multi‑sample analysis | × | https://github.com/zhengli09/BASS | R |
| SpaGCN | graph convolutional network-based model | √ | https://github.com/jianhuupenn/SpaGCN | Python |
| STAGATE | graph attention auto-encoder framework | √ | https://github.com/ttgump/spaVAE/tree/main/src/spaVAE | Python |
| SpatialPCA | Spatially aware dimension reduction | × | https://github.com/shangll123/SpatialPCA. | R |
